# Influence of Environmental Practices on Sustainable Development of the Sub-Saharan African Countries

**DOI:** 10.12688/openresafrica.16443.1

**Published:** 2026-03-25

**Authors:** James C.N Mbugua, Ibrahim Tirimba Ondabu, Fred Ochogo Sporta

**Affiliations:** 1 KCA University, Nairobi, Nairobi County, Kenya; 2 KCA University, Nairobi, Nairobi County, Kenya; 3 KCA University, Nairobi, Nairobi County, Kenya

**Keywords:** Environmental Practices, Sustainable Development, Sub-Saharan Africa, secondary data, System GMM

## Abstract

**Background:**

Although sustainable development has gained global acceptance, there is limited research on the adoption of sustainable development as it stands today, the challenges facing the Sub-Saharan African region and the prospects of sustainable development implementation in the region. This research aimed to determine the influence of environmental practices on sustainable development of the sub-Saharan African countries. The study was guided by the Ecological Modernization Theory.

**Methods:**

The research used a longitudinal design, which was necessary to capture the temporal precedence and had a stronger reason to make a causal conclusion on how the dynamics of the predictor variables result in the dynamics of sustainable development outcomes. The study adopted a positivist research philosophy. The study examined data from 49 Sub-Saharan African countries over 24 years from 2000 to 2023. This period was effectively chosen to embrace a significant longitudinal curve to grasp the short and long-term tendencies in the sustainability-related practices and sustainable development. The study utilized a structured secondary data collection sheet for data collection, designed to explore the multifaceted dimensions of sustainability practices within the Sub-Saharan African region.

**Results:**

System GMM results showed that the Environmental Practices Index (EPI) had a statistically insignificant relationship with sustainable development (β = −0.0329, p = 0.624), while the lagged dependent variable demonstrated strong persistence (β = 0.8124, p < 0.001), leading to the acceptance of the null hypothesis that environmental practices do not influence sustainable development.

**Conclusions:**

The study concluded that current environmental sustainability measures in Sub-Saharan Africa do not significantly influence sustainable development outcomes. The research recommended that future research should examine the implementation-effectiveness gap in sustainability practices, as well as expanding the geographic scope to South Africa, which may produce relationships that differ from those in Latin America, Asia or other regions.

## Background of the study

Sustainable development has emerged as a global priority, driven by the urgent need to address environmental, social and economic challenges ( Ahmed, 2023). Recognizing the importance of achieving balanced progress, the United Nations, in collaboration with world leaders, introduced the 17 SDGs as a blueprint to guide global efforts towards sustainability by 2030 ( Maano & David, 2020). Sustainable development emphasizes the interconnectedness of economic growth, social equity and environmental conservation, ensuring that the present generation meets its needs without compromising the ability of future generations to do the same ( Ying, 2022). This holistic approach underscores the importance of integrating these three pillars to create a more resilient and sustainable future.

The relevance of sustainable development is particularly critical in Sub-Saharan Africa, a region where balancing economic growth, environmental preservation and social well-being presents significant challenges ( Batae et al., 2020). Many countries in the region are striving to address issues such as poverty, inequality and environmental degradation, which complicate their development efforts ( Batae et al., 2020). The complexity of these challenges requires comprehensive strategies that integrate economic, social and environmental considerations. Ogede et al. (2024) highlighted the moderating role of financial inclusion and institutional quality on the relationship between income inequality and carbon emissions, demonstrating how targeted interventions can support sustainable development in the region.

Achieving sustainable development in Sub-Saharan Africa involves navigating diverse challenges and opportunities shaped by legal frameworks, socio-economic contexts and environmental issues. These factors necessitate strategic investments in sustainable national practices and policies. Uduji et al. (2019) examined the role of government initiatives and rural women in sustainable agricultural development, revealing how community engagement and national efforts can drive progress in the Niger Delta region ( Tenaw & Beyene, 2021). Their research illustrates the need for localized strategies that leverage community involvement for achieving sustainable agricultural outcomes. The integration of such approaches is essential for addressing the multifaceted challenges faced by the region ( Batae et al., 2020). Sustainable development requires a coordinated effort from both governmental and non-governmental stakeholders to effectively address the region’s socio-economic and environmental needs ( Batae et al., 2020). Uduji et al. (2019) provide valuable insights into how environmental entrepreneurship, national development strategies and government initiatives can contribute to sustainable development. Investing in these areas is vital for fostering long-term progress and resilience in the Sub-Saharan African region, ensuring that both economic and environmental goals are achieved ( Sun et al., 2020).

The pursuit of sustainable development in Sub-Saharan Africa is further complicated by a range of legal, socio-economic and environmental factors. Governments and stakeholders must navigate these complexities to foster inclusive growth while promoting ecological conservation ( Muigua, 2023). Ojeyinka and Osinubi (2022) explored the impact of financial development on the globalization-sustainable development nexus, noting that robust financial systems are crucial for supporting sustainability initiatives. Additionally, Sarkodie and Adams (2020) examined how factors like electricity access and governance affect income inequality and human development in Sub-Saharan Africa, underscoring the need for integrated strategies that address multiple dimensions of sustainability. To achieve the global SDGs and ensure long-term sustainability, coordinated and multifaceted efforts are essential. The interplay between income inequality, environmental degradation and socio-economic factors necessitates a holistic approach to development. Ogede et al. (2024) emphasized the importance of financial inclusion and institutional quality in moderating the impacts of income inequality and carbon emissions. Meanwhile, Sarkodie and Adams (2020) highlighted how financial development and governance can influence sustainable development outcomes. Addressing these complex issues requires strategic investments, effective governance and collaborative efforts across sectors ( Ogede et al., 2024).

The global community is currently deeply concerned about the sustainability of the planet, which has become a focal point in various significant global initiatives in recent years. Recognizing a pressing requirement for specific criteria to achieve sustainable development, the United Nations and world leaders collaborated to establish the seventeen SDGs to be realized by the year 2030 ( Maano & David, 2020). Sustainable development stands as a critical priority throughout the world, reflecting the collective goals for balanced progress, societal equity and environmental preservation. It embodies a holistic approach that harmonizes economic growth, social welfare and environmental conservation, ensuring the present needs are met without compromising future generations’ abilities to meet their own ( Ying, 2022). The significance of sustainable development extends beyond regional boundaries, acknowledging the intricate interconnectedness between economic prosperity, social well-being and environmental stability ( Zhan, 2023). If nations and key stakeholders do not collaboratively align their endeavours towards the environmental, social and governance facets of sustainability, the realization of the 2030 Agenda for sustainable development will continue to elude us ( Muigua, 2023). In the context of the Sub-Saharan African region, sustainable development is a crucial pillar that encompasses aspirations for comprehensive advancement, inclusive growth and ecological conservation ( Batae et al., 2020). This dedication to sustainable development stems from the need to harmonize economic growth with environmental responsibility and social justice. Sun et al. (2020) discussed the role of environmental entrepreneurship in advancing sustainable development across 35 countries in Sub-Saharan Africa. Their findings underscore the importance of integrating entrepreneurial initiatives with sustainability goals to address the region’s unique challenges. Additionally, Tenaw and Beyene (2021) explored the relationship between environmental sustainability and economic development, providing evidence of a modified Environmental Kuznets Curve (EKC) hypothesis that highlights the complex interplay between economic growth and environmental impacts.

The goals of environmental practices are to reduce the ecological footprint of the countries through the efficient use of resources, decreasing waste, and minimizing the consequences of climate change issues ( Han and Gao, 2024; Naeem et al., 2021). These activities are aimed at conserving ecosystems, improving biodiversity, and switching to low-carbon-based economies. ESG practices focus on environmental sustainability, and these solutions are required to deal with the problems of climate change, the resources depletion and pollution. Reduction of carbon emission, better energy consumption and application of sustainable practices of resource management are gaining interest among both companies and investors ( Agrawal et al., 2023). The environmental aspect of ESG is vital towards reducing the ecological effects and eliminating the sustainability in the long run ( Ahmed, 2023). In a world where many nations are under a serious threat due to climate change and environmental degradation, it is essential to consider environmental implications when developing business strategies to create resilience and contribute to sustainable development ( Agweny, 2021).

### Statement of the problem

While there have been notable global advancements in integrating sustainable practices, which hold great potential for driving sustainable development, Sub-Saharan African countries continue to lag ( Tiony, 2023). According to the United Nations (UN) 2024 Sustainable Development Report, the Sub-Saharan Africa region ranks the lowest compared to other regions such as East and South Asia, Eastern Europe and Central Asia, Latin America and the Caribbean, the Middle East and North Africa and OECD member countries. Mauritius is the top-performing Sub-Saharan African country, ranking 73rd out of 193 United Nations member states. Only two Sub-Saharan African countries, such as Mauritius and Namibia, are among the top 100, with rankings of 73 and 98, respectively.

There is a noticeable gap in the body of knowledge addressing the specific facts in the Sub-Saharan African countries, considering the changing regional and global trends in sustainable development ( Sadiq et al., 2020). Although sustainable development has gained global acceptance, there is limited research on the adoption of sustainable development as it stands today, the challenges facing the Sub-Saharan African region and the prospects of sustainable development implementation in the region ( Cracknell, 2023; Cek, 2023). This knowledge gap underscores the need for a comprehensive examination of sustainable development in the region. Additionally, the absence of studies utilizing advanced econometric analyses within this regional scope suggests a need for more sophisticated analytical techniques to better understand these intricate relationships ( Pawłowska et al., 2022).

### General objective

To determine the influence of environmental practices on sustainable development of the Sub-Saharan African countries.

## Literature review

The intricate relationship between environmental impacts and sustainable development is emphasized by Hoshino and Seixas (2019), advocating for a national development model that integrates ecological integrity, social welfare and economic prosperity. Their study highlighted the need for countries to embed environmental considerations into policy frameworks and decision-making processes to mitigate human-induced environmental damage and support long-term sustainability. These findings align with Shahzad et al. (2020), who examined environmental sustainability and green innovation, finding that embedding environmental practices into national development strategies can yield competitive advantages as public preferences shift toward eco-friendly initiatives. Both studies support the positive impact of environmentally sustainable practices on long-term ecological preservation and sustainable economic positioning.

However, while Hoshino and Seixas (2019) emphasized ecological integrity as a core pillar of sustainable development, Shahzad et al. (2020) extended this argument by highlighting the potential for businesses to access new markets through green innovation. This idea is further supported by Qalati et al. (2023), who demonstrated that environmentally sustainable practices (ESP) could enhance both performance and environmental sustainability. Their research in the Chinese manufacturing sector also identified green employee integration (GEI) as a critical mediator between ESP and performance, indicating that environmental sustainability extends beyond policy integration to include operational and human resource strategies. Although these studies collectively underscore the benefits of environmental practices, Hoshino and Seixas (2019) focus primarily on policy implications, whereas Shahzad et al. (2020) and Qalati et al. (2023) examine the practical benefits for businesses and employees. Despite the consensus on the positive relationship between environmental practices and sustainable development, some studies present nuanced perspectives. Martin and Dahlstrom (2020), for instance, found that stronger environmental policies did not always yield immediate financial gains at the national level, especially in terms of market indicators like stock performance. This contrasts with findings from Shahzad et al. (2020), who argued that countries prioritizing environmental practices see enhanced competitiveness and improved trade opportunities. Martin and Dahlstrom’s (2020) results suggest that the economic benefits of environmental sustainability may vary based on specific economic indicators or market conditions. While the general trend suggests positive outcomes for sustainability-focused nations, the financial returns may not be instantly visible, highlighting the need for a nuanced understanding of how sustainability initiatives impact different aspects of national economic success.

Scholars, including Hoshino and Seixas (2019), Shahzad et al. (2020) and Qalati et al. (2023), all agree on the importance of incorporating environmental practices into sustainable development strategies. However, differences in their focus on policy, business strategy and operational aspects indicate that the relationship between environmental practices and sustainability is multifaceted. These studies collectively highlight the need for businesses and policymakers to adopt a comprehensive approach to environmental sustainability, balancing ecological integrity, competitive market positioning and financial performance. Despite some variations in immediate financial outcomes, the long-term benefits of environmental sustainability are widely recognized across sectors and regions. The relationship between national-level social responsibility initiatives, environmental policies and economic performance was examined by Sarfraz et al. (2023), revealing that CSR-driven policies positively impact both environmental sustainability and economic growth. The study emphasized the role of green innovation and sustainable development as key factors that mediate this relationship, identifying how green innovation strategies strengthen environmental and financial outcomes on a national scale. This finding aligns with Obamen et al. (2021), whose study in Nigeria confirmed the significant positive impact of Environmental Management Practices (EMPs) on sustainable development. Both studies underscore the importance of embedding CSR and EMPs into national strategies to enhance environmental and economic performance, indicating that countries prioritizing these practices can achieve more sustainable outcomes.

However, the findings of Sarfraz et al. (2023) and Obamen et al. (2021) differ in their emphasis. While Sarfraz et al. (2023) stressed the importance of CSR in driving both environmental and financial performance, Obamen et al. (2021) focused on the direct impact of EMP tools on sustainability. The latter study specifically noted that manufacturing firms in Nigeria must possess expertise in EMPs to fully leverage environmental benefits, while Sarfraz et al. (2023) pointed to green innovation as a key driver of performance. These perspectives complement each other by demonstrating how various environmental initiatives, whether framed within CSR or direct EMPs, contribute to sustainable development. Furthermore, both studies reinforce the idea that environmental practices are critical to achieving long-term sustainability, though their specific mechanisms and emphasis on CSR versus EMP tools differ.

Similarly, Tenaw and Beyene (2021) examined the relationship between environmental sustainability and economic development across sub-Saharan African countries, focusing on the modified Environmental Kuznets Curve (EKC) hypothesis. Their study advocated for reducing carbon emissions by investing in renewable energy sources such as solar, wind and hydropower. These findings build on Sarfraz et al. (2023) and Obamen et al. (2021) by suggesting that national policy initiatives, such as decreasing fossil fuel dependency and promoting renewables, are critical to achieving sustainable development. While Sarfraz et al. (2023) and Obamen et al. (2021) emphasized environmental practices, Tenaw and Beyene (2021) broadened the discussion to include the macroeconomic impacts, illustrating how environmental sustainability at the national level supports long-term economic growth. This perspective highlights the importance of a holistic approach to sustainability that incorporates both national policy and organizational practices.

A regional perspective was provided by Bowmans (2022) by analyzing Kenya’s legal and policy frameworks designed to promote environmental sustainability. This study underscored Kenya’s constitutional guarantees of a clean environment, as well as its legislative initiatives, such as the Climate Change Act and the ban on plastic bags. These efforts align with the global sustainability objectives highlighted by Tenaw and Beyene (2021) and Sarfraz et al. (2023), illustrating Kenya’s proactive role in addressing climate change and environmental protection. While Bowmans (2022) emphasized the significance of regulatory frameworks and policies in driving sustainability, Sarfraz et al. (2023) and Obamen et al. (2021) focused more on environmental practices at the corporate level. Nevertheless, all three studies concur that effective environmental management, whether through policy or national initiatives, is crucial for sustainable development. Together, the studies by Sarfraz et al. (2023), Obamen et al. (2021), Tenaw and Beyene (2021) and Bowmans (2022) provide a comprehensive perspective on the impact of environmental practices on sustainable development. Sarfraz et al. and Obamen underscore the role of CSR and EMPs at the organizational level, while Tenaw and Beyene advocate for policy-driven sustainability measures. Bowmans adds a regional dimension, highlighting how regulatory frameworks can help countries like Kenya achieve sustainability objectives. Collectively, these studies underscore the need for policy initiatives to ensure long-term environmental sustainability.

### Theoretical framework

The study used the Ecological Modernization Theory. Theorists, such as Joseph Huber and Martin Janicke ( Mol, SpAargaren & Sonnenfeld, 2020), developed a new environmental sustainability paradigm, calling it Ecological Modernization Theory (EMT). As opposed to the classical theories on the environment, where in most of the cases, the development of industrial growth is looked at as an inevitably degrading process, EMT states that economic growth and technology can coexist and, in fact, lead to environmental sustainability. This theory suggests that changes in the regulations and structural changes in society are certain to make societies modernize with the assistance of technological innovation that offers these changes. EMT offers the formula of those industrialized nations, which may theoretically lower their ecological imprint without worsening the economic development of the state through stimulating the use of clean technologies, sustainable use of resources, and green economy policy ( Julkovski et al., 2021). This makes the theory particularly relevant in Sub-Saharan African nations, where sustainable development is of significant relevance in economic development, given the fact that the region has been reliant on natural resources and is also prone to environmental degradation.

Among the key arguments of EMT, it should be noted that technological innovation and environmental policy reforms represent the key elements of the sustainability development realization. The theory suggests that in the development of clean technology, such as renewable energy, waste management system and resource-efficient manufacturing are possible, which will help to mitigate the damage to the environment without decelerating the growth of an economy. According to Bugden (2022), such a form of innovation provides a possibility to divide the sphere of economic activity and environmental degradation, therefore enabling countries to follow both economic and environmental goals. Moreover, EMT also emphasizes that regulatory frameworks and market-based incentives could also assist businesses and industries in utilizing sustainable practices. Such policies include the environmental taxes, the emissions trading and the subsidies on the clean technologies, which create a business case in favour of environmental responsibility, and this is the reason the clean practices should be invested in ( Xie et al., 2022). Such policies can encourage investments in green technology within the Sub-Saharan African setup that would precipitate economic growth and safeguard the environment.

EMT ontologically assumes that environmental problems are not natural problems but problems which are deeply rooted in the social and economic systems. Under this outlook, environmental concerns have a combination effect on industrial development and economic processes, and the problems should be addressed in a coordinated manner, not as individual actions ( Mol et al., 2020). EMT, in epistemological terms, is pragmatic and relies on empirical studies and policy research in order to come up with effective mechanisms that can be used to achieve a balance between the development of the economy and environmental sustainability. The theory challenges policymakers and scholars to find solutions that add to the existing economic systems as they transform into sustainable ones. This epistemological approach is applicable in the Sub-Saharan Africa scenario, where pragmatic and responsive solutions are needed because of the disparity in industrial development and environmental problems in the area.

Despite the positivity, critics argue that EMT is too optimistic about the future of the technological and regulatory solutions, and at times it underestimates the level of behavioural and cultural changes to have the actual sustainability ( Julkovski et al., 2021). Besides, the reliance of EMT on the existing technological advancement assumes the resources and infrastructures, which cannot necessarily be the case in the developing nations, including Sub-Saharan Africa. Another point that the theory has been criticized for is its focus on top-down, state-based approaches that do not give significant attention to the contribution of grassroots movements and the involvement of local communities in the process of environmental change. It is even more so in Sub-Saharan Africa, where local knowledge and community participation on most occasions is the secret of success of any environmental project (Mol, 2001). Besides, there is the possibility that EMT will not fully offset the imbalance of power that leads to environmental exploitation since the corporations and the richer countries will have higher control over the policy-making process than the developing nations.

Based on the example of Sub-Saharan Africa, EMT introduces a valid framework for reflecting on viable aspects of the economic development plans of the region with respect to sustainable practices. The development of technology and green investments should be part of the policies that African governments adopt so that they can not only realize economic growth but also make sure that such issues as deforestation, lack of water and air pollution become a thing of the past. To illustrate the point, the renewable energy projects and green infrastructure development have already started to be embraced in such states as Kenya and South Africa, which aligns with the principles of EMT ( Salifu and Salifu, 2024). These ventures reduce the environmental effects, in addition to offering jobs and financial security. EMT further indicates that there can be a partnership between governments, the private sector, and civil society organizations to enable sustainable development. Through collaboration, these stakeholders will be in a position to share resources and skills in order to develop contextual solutions to environmental sustainability. Moreover, the emphasis of EMT on policy changes and market incentives is closely connected with the interest of Sub-Saharan Africa in environmental regulations because the countries of the region strive to find a balance between economic growth and environmental sustainability.

## Methodology

This study used a longitudinal panel design and incorporated both the descriptive and explanatory elements that looked at sustainability dynamics in the Sub-Saharan African region within the 24-year period in a comprehensive manner. The descriptive element of the design was applied to capture in a systematic manner and to document the nature, trend and current situation of the sustainability practices, institutional quality and sustainable development in the region and over time. Simultaneously, the dynamic relations and causal inferences between the independent and dependent variables were examined using the explanatory component. Precisely, the research aimed at providing an explanation of the joint effects of sustainability practices and institutional quality on sustainable development outcomes over time.

The study adopted a positivist research philosophy to investigate the relationships between sustainability practices, institutional quality and sustainable development in Sub-Saharan African countries. Grounded in the belief that reality was objective and measurable, positivism emphasized empirical evidence, hypothesis testing, and statistical analysis.

The study examined data from 49 Sub-Saharan African countries over a 24-year period from 2000 to 2023 to analyze sustainability practices, institutional quality and their influence on sustainable development. The period after 2000 is a milestone in the history of the region, with a rise in attention to sustainability in the world and the adoption of the SDGs in 2015 and the development of more ESG-related policies and investment trends in the emerging economies ( United Nations, 2015; World Bank, 2023).

The study relied exclusively on secondary data from reputable sources, including the World Bank Data Bank (2025), UNDP (2025), Fund for Peace (2025) and Sustainable Development Report (2024). The World Bank provided data on environmental indicators like greenhouse gas emissions, renewable energy, and forest cover, alongside governance metrics such as corruption and government effectiveness indices. The UNDP supplied data on social rights, gender equality, and child labour from Human Development Reports. The Fragile States Index, acquired by the Fund for Peace, was used as the measurement of institutional quality. No permissions were needed as data came from open-access or licensed sources accessed via institutional subscriptions.

After data cleaning, descriptive statistics, that is, standard deviations and means, were conducted for each variable. Data analysis encompassed the use of descriptive statistics and regression models. Stata Version 15 was employed for the analysis, ensuring sophisticated analysis and interpretation of the data. The data was then presented in tables. The resulting GMM model equation, as by Blundell and Bond (1998) are below;

$$Y_{\mathrm{it}}=\beta_{0}+\beta_{1}Y_{1\mathrm{it}-1}+\sum_{k=1}^{K}\beta_{2k}{\mathrm{ENV}}_{k,\mathrm{it}}+\mu_{\mathrm{it}}+\epsilon_{\mathrm{it}}$$ (3.1a)

$$Y_{\mathrm{it}}=\beta_{0}+\beta_{1}Y_{1\mathrm{it}-1}+\sum_{k=1}^{K}\beta_{2k}{\mathrm{ENV}}_{k,\mathrm{it}}+{\mathrm{CV}}_{\mathrm{it}}+\mu_{\mathrm{it}}+\epsilon_{\mathrm{it}}$$ (3.1b)

Where;

$Y_{\mathrm{it}}\;$ = Dependent Variables over time period.

$\;Y_{1\mathrm{it}-1}$ = One time period Lag of the dependent variables.

$\sum_{k=1}^{K}F_{k,\mathrm{it}}\;$ = Is a set of one Independent variable indicator.

${\mathrm{INV}}_{k,\mathrm{it}},{\mathrm{SOC}}_{k,\mathrm{it}},{\mathrm{GOV}}_{k,\mathrm{it}}\;\text{and}\;{\mathrm{ECO}}_{k,\mathrm{it}}$ = Independent variables over time period.

$\beta_{0}$ = Intercept.

$\beta_{1}-\beta_{2}\;$ = Slope of Coefficients.

${\mathrm{CV}}_{\mathrm{it}}$ = Control variables over time period.

$\mu_{\mathrm{it}}$ = Country-specific effects over time period.

$\epsilon_{\mathrm{it}}$ = Error term over time period.

## Results and discussion

### General information

The study utilized secondary data from 49 Sub-Saharan African countries covering the period 2000–2023. The data were a structured balanced panel, with annual observations across the 14-year period. Initially, the dataset consisted of 1,176 observations based on 49 SSA countries, but was reduced to 1,080 as Eritrea, Eswatini (formerly Swaziland), Somalia and South Sudan were deleted due to missing data. All indicators were measured annually and standardized to indices to ensure comparability across countries and over time. The dataset captured environmental, social, governance, and economic practices, alongside financial development, institutional quality, and sustainable development outcomes. Table 1 presents a summary of the dataset characteristics.

**Table 1.** General information on study data.

Indicator	Description	Coverage
Countries Included	Sub-Saharan African countries	45
Study Period	Time frame of data collected	24 years (2000–2023)
Unit of Analysis	Country-level annual observations	Panel data
Number of Observations	Total dataset size	1,080 (45 × 24 years)
Panel Structure	Dataset structure	Strongly balanced panel
Measurement Periodicity	Frequency of data collection	Annual
Data Type	Nature of data used	Secondary quantitative and qualitative document analysis

### Descriptive statistics

This section presents the descriptive statistics of the study variables, including environmental practices, social practices, governance practices, economic practices, financial development, institutional quality, and sustainable development, for the 45 Sub-Saharan African countries over the period 2000–2023. The results summarize the distribution of the variables in terms of their mean and standard deviation, providing an overview of central tendencies and variations across countries and years.

The descriptive statistics for environmental indicators showed that the GHG Emissions Index had the highest mean (M = 0.894, SD = 0.148), reflecting generally favourable emissions performance across SSA, with relatively low variation among countries. The Renewable Energy Consumption Index recorded a moderate average (M = 0.644, SD = 0.268), suggesting uneven adoption of renewable energy across the region. The Forest Area Cover Index posted the lowest mean (M = 0.326, SD = 0.252), indicating limited forest coverage with substantial variation among countries. These results highlight that while emissions levels are relatively low, renewable energy adoption is moderate, and forest conservation remains a significant regional challenge, underscoring persistent environmental sustainability concerns.

The descriptive statistics for sustainable development measures showed mixed progress with positive environmental adjustments. The Human Development Index recorded a moderate average (M = 0.517, SD = 0.112), reflecting ongoing development challenges. The Adjusted Net Savings Index showed reasonable performance (M = 0.571, SD = 0.128). The Child Mortality Index reflected substantial health improvements (M = 0.840, SD = 0.096), though with remaining disparities between countries. These results present a portrait of moderate development progress that is constrained by persistent structural challenges ( Table 2).

**Table 2.** Descriptive statistics for the variables.

Variable	Obs	Mean	Std. Dev.
ghg_index	1,080	0.894	0.148
ren_en_index	1,080	0.644	0.268
forest_cov_index	1,080	0.326	0.252
hdi_index	1,080	0.517	0.112
nat_save_index	1,080	0.571	0.128
mort_ind_index	1,080	0.840	0.096

Note: All indices normalized to 0–1 scale, where higher values indicate better performance.

The observed pattern of relatively strong emissions performance coupled with forest conservation challenges reflects documented regional characteristics. Sub-Saharan Africa’s per capita CO _2_ emissions are low ( World Bank, 2023). Conversely, the FAO (2020) reports the region accounted for the highest net loss of forest area of any region between 2010 and 2020, identifying it as a primary deforestation hotspot.

### Panel unit root tests and stationarity tests

Environmental indicators such as the GHG Emissions Index (ghg_index) and the Renewable Energy Consumption Index (ren_en_index) were strongly stationary. The results for the variables confirm they meet the critical assumption of stationarity required for subsequent panel regression analysis ( Table 3).

**Table 3.** Levin-Lin-Chu unit-root test for the variables.

Variable	Unadjusted t	Adjusted t*	p-value
ghg_index	−15.7913	−5.2572	0.0000
ren_en_index	−14.1928	−5.6037	0.0000
forest_cov_index	−8.5448	−3.4510	0.0003
hdi_index	−9.4484	−2.4561	0.0070
nat_save_index	−8.1004	−8.1004	0.0000
mort_ind_index	−34.3783	−30.6473	0.0000

Note: H _0_: Panel contains unit roots (non-stationary).

The Fisher-type panel unit root test results, presented in Table 4, indicate that GHG Emissions Index (ghg_index) demonstrate strong evidence of stationarity. The Renewable Energy Consumption Index (ren_en_index) and Forest Area Cover Index (forest_cov_index) show evidence of non-stationarity, with both tests failing to reject the null hypothesis (p > 0.05). The findings are shown in Table 4.

**Table 4.** Unit root test using ADF and PP (Fisher type) tests.

Variable	ADF test	p-value	PP test	p-value
ghg_index	2.6786	0.0037	2.8060	0.0025
ren_en_index	0.0162	0.4935	−0.9599	0.8314
forest_cov_index	−2.5829	0.9951	−3.1866	0.9993
hdi_index	−1.0158	0.8451	−1.4473	0.9261
nat_save_index	5.2035	0.0000	18.8535	0.0000
mort_ind_index	38.8301	0.0000	17.6178	0.0000

Note: H _0_: Panel contains unit roots (non-stationary).

The results of the Fisher-type panel unit root tests on the first-differenced series, presented in Table 5, demonstrate that the transformation successfully achieved stationarity for both previously non-stationary variables. For each differenced series, both the Augmented Dickey-Fuller (ADF) and Phillips-Perron (PP) tests decisively reject the null hypothesis of non-stationarity at the 1% significance level (p = 0.0000).

**Table 5.** First differencing using ADF and PP (Fisher type) tests.

Variable	ADF test	p-value	PP test	p-value
d_ren_en_index	40.6475	0.0000	182.7248	0.0000
d_forest_cov_index	42.4784	0.0000	210.7608	0.0000
d_hdi_index	30.0216	0.0000	211.6077	0.0000

Note: H _0_: Panel contains unit roots (non-stationary).

The exceptionally large test statistics across all variables provide strong evidence of stationarity. The Forest Area Cover Index (d_forest_cov_index) exhibited the most substantial evidence of stationarity in the PP tests, with statistics of 210.76. Similarly, the Renewable Energy Consumption Index (d_ren_en_index) showed robust stationarity with an ADF statistic of 40.65 and a PP statistic of 182.72.

These results confirm that the first-difference transformation effectively eliminated the unit roots present in the original level series. Consequently, all two differenced variables, including d_ren_en_index and d_forest_cov_index, are now integrated of order zero, I(0) and meet the stationarity assumption required for valid panel regression analysis. The transformation enables their inclusion alongside the nine originally stationary variables in subsequent econometric modelling without spurious regression concerns.

### Multicollinearity test

The Variance Inflation Factor (VIF) analysis confirms the absence of harmful multicollinearity among all independent variables across the different model specifications. For every variable group, both individual and mean VIF values are substantially below the standard critical threshold of 10, with most falling well below the more conservative threshold of 5. Environmental practices demonstrate minimal multicollinearity with a mean VIF of 1.03 and individual values of 1.02–1.04. These results provide strong evidence that the explanatory variables are not excessively correlated, ensuring that the parameter estimates in subsequent regression analysis will be stable, reliable, and interpretable. The data is therefore robust and suitable for multivariate panel estimation without multicollinearity concerns ( Table 6).

**Table 6.** Multicollinearity test results.

DV	Independent variables	VIF	1/VIF	Mean VIF
SD	ghg_index, d_ren_en_index, d_forest_cov_index	1.02, 1.03, 1.04	0.978, 0.969, 0.958	1.03

### System GMM model diagnostics

The system GMM regression results reveal significant insights into the determinants of sustainable development (SD) across Sub-Saharan Africa. The lagged dependent variable (L.SD) demonstrates a strong positive and statistically significant coefficient (β = 0.593, p < 0.001), indicating substantial persistence in sustainable development outcomes. This suggests that previous levels of sustainable development strongly influence current performance, with approximately 59% of prior SD levels carrying forward to the current period. This finding underscores the path-dependent nature of sustainable development, where past achievements create momentum for future progress.

Among environmental practices, none of the variables achieved statistical significance at conventional levels. The GHG Emissions Index (β = 0.103, p = 0.141), Renewable Energy Consumption Index (β = 0.047, p = 0.672), and Forest Area Cover Index (β = −6.466, p = 0.163) all showed statistically insignificant relationships with sustainable development. This suggests that, in the comprehensive model accounting for all other factors, individual environmental indicators may not directly drive sustainable development improvements or their effects may be mediated through other channels ( Table 7).

**Table 7.** Two-Step system GMM estimation results.

Variable	Coefficient (β)	Std. Error	t-statistic	p-value	95% Confidence Interval
**Lagged Dependent Variable**					
L.SD	0.5933	0.0946	6.27	0.000***	(0.4078, 0.7789)
**Environmental Practices**					
ghg_index	0.1030	0.0700	1.47	0.141	(−0.0342, 0.2403)
d_ren_en_index	0.0468	0.1104	0.42	0.672	(−0.1697, 0.2633)
d_forest_cov_index	−6.4661	4.6393	−1.39	0.163	(−15.5589, 2.6266)

The model diagnostics confirm the validity of the estimation approach. The Arellano-Bond tests show appropriate serial correlation patterns, with significant AR (1) (p = 0.007) and insignificant AR (2) (p = 0.798), satisfying the key assumption for GMM estimation ( Table 8).

**Table 8.** Arellano-Bond test for serial correlation.

Test	z-statistic	p-value	Conclusion
AR (1) in first differences	−2.69	0.007	Serial correlation present
AR (2) in first differences	0.26	0.798	No serial correlation

The Hansen J-test of overidentifying restrictions showed a χ ^2^ statistic of 24.49 with 974 degrees of freedom and a p-value of 1.000, failing to reject the null hypothesis that the instruments are valid. This non-significant result confirms the validity of the instrument set used in the system GMM estimation. The Difference-in-Hansen test for the GMM instruments for levels yielded a χ ^2^ statistic of −7.83 with 94 degrees of freedom and a p-value of 1.000, indicating that the exogeneity assumption for this instrument subset cannot be rejected. These diagnostics confirm the appropriateness of the model specification and the reliability of the instruments used ( Table 9).

**Table 9.** Instrument validity by Hansen J-test and Difference-in-Hansen test.

Test	χ ^2^ statistic	df	p-value	Conclusion
Hansen J-test of overid. Restrictions	24.49	974	1.000	Instruments valid
Difference-in-Hansen (GMM instruments for levels)	−7.83	94	1.000	Exogeneity not rejected

The White’s test was conducted to test the null hypothesis (H _0_) that the regression model exhibits homoskedasticity (constant variance of the error term). The results provide strong evidence to reject the null hypothesis of homoskedasticity at the 1% significance level. This conclusion is based on the statistically significant chi-squared statistic for the heteroskedasticity component (χ ^2^ (135) = 268.70, p < 0.001).

The decomposition of the IM-test reveals distinct patterns in the error structure. While the heteroskedasticity component is highly significant (p < 0.001), the skewness component is not statistically significant at conventional levels (χ ^2^ (15) = 15.12, p = 0.443), indicating that the distribution of residuals is approximately symmetric. However, the kurtosis component shows statistical significance (χ ^2^ (1) = 4.31, p = 0.038), suggesting some deviation from normal distribution in the tails of the residual distribution. The combined total test statistic (χ ^2^ (151) = 288.12, p < 0.001) confirms the overall presence of non-spherical errors. These results indicate that while the residuals are symmetrically distributed, they exhibit heteroskedasticity and some non-normal kurtosis. This finding validates the use of robust standard errors in the GMM estimation to address the heteroskedasticity concern and ensure reliable inference despite the non-normal error distribution characteristics ( Table 10).

**Table 10.** White’s Test for Heteroskedasticity.

Test component	Chi ^2^	df	P-value
Heteroskedasticity	268.70	135	0.0000
Skewness	15.12	15	0.4430
Kurtosis	4.31	1	0.0380
Total	288.12	151	0.0000

Before model estimation, a comprehensive series of diagnostic tests was conducted to ensure the robustness and validity of the empirical strategy. Panel unit root tests confirmed the stationarity of the transformed series. Tests for multicollinearity indicated that the explanatory variables were not highly correlated, mitigating concerns of inflated variances. Critically, the specification of the dynamic panel model was validated: the Arellano-Bond test confirmed the absence of second-order serial correlation in the differenced errors, and the Hansen J-test supported the validity of the instrument set. With all key econometric assumptions satisfied, the study proceeded to estimate the System GMM models to test the formulated hypotheses and draw causal inferences.

### System GMM model

The system GMM results for the hypothesis indicate that the Environmental Practices Index (EPI) has a statistically insignificant relationship with sustainable development (β = −0.0329, p = 0.624). The lagged dependent variable shows strong persistence (β = 0.8124, p < 0.001), explaining approximately 81% of current sustainable development levels, as shown in Table 11. This finding suggests that, contrary to the theoretical expectations established in the literature review ( Hoshino & Seixas, 2019; Shahzad et al., 2020; Qalati et al., 2023), current environmental sustainability measures in Sub-Saharan Africa do not translate into measurable improvements in composite sustainable development outcomes. The negative, albeit insignificant, coefficient even hints at potential misalignment or inefficiencies in how environmental policies are implemented or integrated within broader development frameworks in the region. This empirical result provides a critical counterpoint to studies like Obamen et al. (2021) and Sarfraz et al. (2023), which reported positive relationships, suggesting that the institutional, economic, or implementation context in SSA may disrupt the theoretical link between environmental stewardship and holistic development.

**Table 11.** Environmental practices and sustainable development.

Variable	Coefficient (β)	Std. Error	z-statistic	p-value	95% Confidence Interval
**Lagged Dependent Variable**					
L.SDI	0.8124	0.0218	37.27	0.000***	(0.7697, 0.8552)
EPI	−0.0329	0.0670	−0.49	0.624	(−0.1643, 0.0985)
_cons	0.1019	0.0208	4.90	0.000***	(0.0611, 0.1428)

H _01_: Environmental practices do not influence the sustainable development of the Sub-Saharan African countries.

The lagged dependent variable shows strong and highly significant persistence, explaining significant variation in current sustainable development levels. This overwhelming path dependence is a crucial finding. It indicates that sustainable development in SSA is predominantly determined by its own historical trajectory, creating a high degree of inertia. This result aligns with Martin and Dahlstrom’s (2020) view that the benefits of environmental sustainability may not be immediate and could be overshadowed by entrenched structural factors. The strong autoregressive effect suggests that breaking from historical development paths to incorporate new sustainability paradigms is exceptionally challenging, potentially explaining why current environmental practices fail to show a significant independent impact.

This empirical outcome necessitates a critical re-evaluation of the policy frameworks discussed by Bowmans (2022) and the implementation mechanisms highlighted by Agweny (2021). While countries like Kenya have established progressive environmental legislation, such as the Climate Change Act and plastic bag bans, and sectors like freight transport show cost benefits from green practices, these isolated successes do not aggregate to a significant macroeconomic relationship at the regional level. The findings suggest that environmental practices in SSA may be either too fragmented, inadequately scaled, or insufficiently integrated with other development dimensions to produce measurable impacts on the composite sustainable development index. This supports Ying’s (2022) sector-specific findings, suggesting that environmental benefits may be context-dependent and not automatically translate to national or regional development metrics.

The Arellano-Bond test results for the Environmental Practices model show appropriate serial correlation patterns for valid GMM estimation. The significant AR (1) test (z = −2.52, p = 0.012) indicates first-order serial correlation in the first-differenced errors, which is expected and acceptable in dynamic panel models. The insignificant AR (2) test (z = 1.09, p = 0.275) confirms the absence of second-order serial correlation, satisfying the key assumption that the error term in the level equation is not serially correlated. This pattern validates the moment conditions and supports the reliability of the GMM estimator ( Table 12).

**Table 12.** Arellano-Bond test for serial correlation for environmental practices model.

Test	z-statistic	p-value	Conclusion
AR(1) in first differences	−2.52	0.012	Serial correlation present
AR(2) in first differences	1.09	0.275	No serial correlation

The instrument validity tests confirm the appropriateness of the instrument set used in the Environmental Practices GMM estimation. The Hansen J-test yields a χ ^2^ statistic of 43.87 with 525 degrees of freedom and a p-value of 1.000, failing to reject the null hypothesis that the instruments are valid. The Difference-in-Hansen test for the GMM instruments for levels shows a χ ^2^ statistic of −0.90 with 43 degrees of freedom and a p-value of 1.000, indicating that the exogeneity assumption for this instrument subset cannot be rejected. These results collectively support the validity of the instruments ( Table 13).

**Table 13.** Instrument validity tests for environmental practices model.

Test	χ ^2^ statistic	df	p-value	Conclusion
Hansen J-test of overid. Restrictions	43.87	525	1.000	Instruments valid
Difference-in-Hansen (GMM instruments for levels)	−0.90	43	1.000	Exogeneity not rejected

## Conclusion

The study concludes that current environmental sustainability measures in Sub-Saharan Africa do not significantly influence sustainable development outcomes. The statistically insignificant relationship between the Environmental Practices Index and sustainable development suggests that environmental policies and initiatives in the region may not be effectively designed, implemented or integrated to meaningfully advance broader sustainable development goals. 

Existing environmental policies require substantive enhancement through better integration and implementation mechanisms. The insignificant relationship between environmental practices and sustainable development suggests that current policies may be inadequately designed or poorly enforced. Policymakers should develop integrated environmental sustainability frameworks that explicitly link specific environmental interventions such as emissions control, renewable energy adoption and forest conservation to measurable sustainable development outcomes, with clear monitoring and evaluation systems to track progress and impact.

## Software availability

https://www.stata.com/stata16/. All analyses were conducted using STATA Version 16.

## Ethics and consent

Ethical approval and consent were not required.
