# Influence of Environmental Practices on Sustainable Development of the Sub-Saharan African Countries

**DOI:** 10.12688/openresafrica.16443.2

**Published:** 2026-07-07

**Authors:** James C.N Mbugua, Ibrahim Tirimba Ondabu, Fred Ochogo Sporta

**Affiliations:** 1 KCA University, Nairobi, Nairobi County, Kenya; 2 KCA University, Nairobi, Nairobi County, Kenya; 3 KCA University, Nairobi, Nairobi County, Kenya

**Keywords:** Environmental Practices, Sustainable Development, Sub-Saharan Africa, secondary data, System GMM

## Abstract

**Background:**

While sustainable development has garnered widespread international support, there is a notable gap in research concerning the practical obstacles and opportunities associated with implementing sustainable development in Sub-Saharan Africa. This research aimed to determine the influence of environmental practices on sustainable development of the sub-Saharan African countries. The study was guided by the Ecological Modernization Theory.

**Methods:**

The research used a longitudinal panel design, which was necessary to capture the temporal precedence and provided a stronger basis for causal inference. The study adopted a positivist research philosophy. The study examined data from 49 Sub-Saharan African countries over 24 years from 2000 to 2023. This period was effectively chosen to embrace a significant longitudinal curve to grasp the short and long-term tendencies in the sustainability-related practices and sustainable development. The study utilized a structured secondary data collection sheet for data collection, designed to explore the multifaceted dimensions of environmental practices, including GHC emissions, renewable energy and forest area cover within the Sub-Saharan African region.

**Results:**

System GMM results showed that the Environmental Practices Index (EPI) had a statistically insignificant relationship with sustainable development (β = −0.0329, p = 0.624), while the lagged dependent variable demonstrated strong persistence (β = 0.8124, p < 0.001), leading to the acceptance of the null hypothesis that environmental practices do not influence sustainable development.

**Conclusions:**

The study concluded that current environmental sustainability measures in Sub-Saharan Africa do not significantly influence sustainable development outcomes, implying that policy efforts must prioritize institutional capacity and enforcement mechanisms over mere regulatory adoption. The research recommended that future research should examine the implementation-effectiveness gap in environmental practices, as well as expanding the geographic scope to South Africa, which may produce relationships that differ from those in Latin America, Asia or other regions.

## Background of the study

Sustainable development has emerged as a global priority, driven by the urgent need to address environmental challenges such as sues such as poverty, inequality and environmental degradation ( Maano & David, 2020; Ahmed, 2023). Achieving sustainable development in Sub-Saharan Africa involves navigating diverse challenges and opportunities shaped by legal frameworks, socio-economic contexts and environmental issues. Uduji et al. (2019) examined the role of government initiatives and rural women in sustainable agricultural development, revealing how community engagement and national efforts can drive progress in the Niger Delta region ( Tenaw & Beyene, 2021). Their research illustrates the need for localized strategies that leverage community involvement for achieving sustainable agricultural outcomes. The integration of such approaches is essential for addressing the multifaceted challenges faced by the region ( Batae et al., 2020). Uduji et al. (2019) provide valuable insights into how environmental entrepreneurship, national development strategies and government initiatives can contribute to sustainable development. Investing in these areas is vital for fostering long-term progress and resilience in the Sub-Saharan African region, ensuring that both economic and environmental goals are achieved ( Sun et al., 2020).

Governments and stakeholders must navigate these complexities to foster inclusive growth while promoting ecological conservation ( Muigua, 2023). Ojeyinka and Osinubi (2022) explored the impact of financial development on the globalization-sustainable development nexus, noting that robust financial systems are crucial for supporting sustainability initiatives. Additionally, Sarkodie and Adams (2020) examined how factors like electricity access and governance affect income inequality and human development in Sub-Saharan Africa, underscoring the need for integrated strategies that address multiple dimensions of sustainability. The interplay between income inequality, environmental degradation and socio-economic factors necessitates a holistic approach to development.

Failure of nations and stakeholders to align efforts across environmental, social, and governance dimensions will undermine the achievement of the 2030 Sustainable Development Agenda ( Muigua, 2023; Zhan, 2023; Ogede et al., 2024)). Sun et al. (2020) discussed the role of environmental entrepreneurship in advancing sustainable development across 35 countries in Sub-Saharan Africa. Their findings underscore the importance of integrating entrepreneurial initiatives with sustainability goals to address the region’s unique challenges. Additionally, Tenaw and Beyene (2021) explored the relationship between environmental sustainability and economic development, providing evidence of a modified Environmental Kuznets Curve (EKC) hypothesis that highlights the complex interplay between economic growth and environmental impacts.

The goals of environmental practices are to reduce the ecological footprint of the countries through the efficient use of resources, decreasing waste, and minimizing the consequences of climate change issues ( Han and Gao, 2024; Naeem et al., 2021). ESG practices focus on environmental sustainability, and these solutions are required to deal with the problems of climate change, the resource depletion and pollution. Reduction of carbon emission, better energy consumption and application of sustainable practices of resource management are gaining interest among both companies and investors ( Agrawal et al., 2023). The environmental aspect of ESG is crucial for ensuring long term sustainability and reducing ecological impacts ( Ahmed, 2023). In a world where many nations are under a serious threat due to climate change and environmental degradation, it is essential to consider environmental implications when developing business strategies to create resilience and contribute to sustainable development ( Agweny, 2021).

### Statement of the problem

While there have been notable global advancements in integrating sustainable practices, which hold great potential for driving sustainable development, Sub-Saharan African countries continue to lag ( Tiony, 2023). According to the United Nations (UN) 2024 Sustainable Development Report, the Sub-Saharan Africa region ranks the lowest compared to other regions such as East and South Asia, Eastern Europe and Central Asia, Latin America and the Caribbean, the Middle East and North Africa and OECD member countries. Mauritius is the top-performing Sub-Saharan African country, ranking 73rd out of 193 United Nations member states. Only two Sub-Saharan African countries, such as Mauritius and Namibia, are among the top 100, with rankings of 73 and 98, respectively.

There is a noticeable gap in the body of knowledge addressing the specific sustainability issues in Sub-Saharan African countries, considering the changing regional and global trends in sustainable development ( Sadiq et al., 2020). Although sustainable development has gained global acceptance, there is limited research on the adoption of sustainable development as it stands today, the challenges facing the Sub-Saharan African region and the prospects of sustainable development implementation in the region ( Cracknell, 2023; Cek, 2023). This highlights the need for a comprehensive regional analysis and the application of advanced econometric techniques to better capture the complex relationships in Sub-Saharan Africa ( Pawłowska et al., 2022).

### Research gap and contribution

Building on these gaps, this study contributes to the literature in three key ways. First, it provides updated empirical evidence on the influence of environmental practices on sustainable development using a large panel of 49 Sub-Saharan African countries over a 24-year period (2000–2023), thereby addressing the limited regional coverage in existing studies and the tendency of prior research to focus on developed or emerging economies with stronger institutional environments. Second, it applies a dynamic System GMM estimation technique, which allows for the control of endogeneity, unobserved heterogeneity, and persistence in sustainable development outcomes, thus strengthening methodological rigor compared to prior studies that rely mainly on static models, and making it particularly suitable for the African context where reverse causality and structural heterogeneity are prevalent. Third, from a theoretical perspective, the study extends the Ecological Modernization Theory (EMT) and ESG discourse by testing their applicability in a context where ecological modernization is still emerging, thereby providing evidence on the extent to which environmental reforms translate into measurable development outcomes under weak institutional settings. Finally, the study’s non-significant findings are theoretically important as they challenge the implicit assumption within EMT and ESG literature that environmental practices automatically translate into sustainability gains, showing instead that in Sub-Saharan Africa, structural and institutional constraints may delay or weaken this transition.

### General objective

To determine the influence of environmental practices on sustainable development of the Sub-Saharan African countries.

## Literature review

The intricate relationship between environmental impacts and sustainable development is emphasized by Hoshino and Seixas (2019), advocating for a national development model that integrates ecological integrity, social welfare and economic prosperity. These findings align with Shahzad et al. (2020), who examined environmental sustainability and green innovation, finding that embedding environmental practices into national development strategies can yield competitive advantages as public preferences shift toward eco-friendly initiatives. Both studies support the positive impact of environmentally sustainable practices on long-term ecological preservation and sustainable economic positioning. This consensus across diverse geographical contexts suggests a theoretical convergence, yet it raises an unanswered question: do these positive associations hold in weak institutional environments where policy implementation capacity is limited, such as Sub-Saharan Africa?

However, while Hoshino and Seixas (2019) emphasized ecological integrity as a core pillar of sustainable development, Shahzad et al. (2020) extended this argument by highlighting the potential for businesses to access new markets through green innovation. This idea is further supported by Qalati et al. (2023), who demonstrated that environmentally sustainable practices (ESP) could enhance both performance and environmental sustainability. Despite the consensus on the positive relationship between environmental practices and sustainable development, some studies present nuanced perspectives. Martin and Dahlstrom (2020), for instance, found that stronger environmental policies did not always yield immediate financial gains at the national level, especially in terms of market indicators like stock performance.

This contrasts with findings from Shahzad et al. (2020), who argued that countries prioritizing environmental practices see enhanced competitiveness and improved trade opportunities. Martin and Dahlstrom’s (2020) results suggest that the economic benefits of environmental sustainability may vary based on specific economic indicators or market conditions. While the general trend suggests positive outcomes for sustainability-focused nations, the financial returns may not be instantly visible, highlighting the need for a nuanced understanding of how sustainability initiatives impact different aspects of national economic success. The contradiction reveals a conceptual distinction that most studies overlook: the difference between short-term financial returns and long-term sustainability outcomes. This distinction implies that the relationship between environmental practices and sustainable development may be temporally contingent, with benefits materializing only after extended time horizons that panel data models must explicitly accommodate.

This finding aligns with Obamen et al. (2021), whose study in Nigeria confirmed the significant positive impact of Environmental Management Practices (EMPs) on sustainable development. Both studies underscore the importance of embedding CSR and EMPs into national strategies to enhance environmental and economic performance, indicating that countries prioritizing these practices can achieve more sustainable outcomes. However, the findings of Sarfraz et al. (2023) and Obamen et al. (2021) differ in their emphasis. While Sarfraz et al. (2023) stressed the importance of CSR in driving both environmental and financial performance, Obamen et al. (2021) focused on the direct impact of EMP tools on sustainability. The latter study specifically noted that manufacturing firms in Nigeria must possess expertise in EMPs to fully leverage environmental benefits, while Sarfraz et al. (2023) pointed to green innovation as a key driver of performance. This divergence suggests that environmental practices operate through multiple channels, including policy instruments, firm-level capabilities, and innovation systems, which are not consistently captured across studies. A key conceptual gap lies in the lack of integration between CSR-driven approaches and technical EMP-based frameworks within a unified sustainability model.

Similarly, environmental sustainability and economic development have been linked across sub-Saharan African countries ( Tenaw & Beyene, 2021), advocating for reduced carbon emissions by investing in renewable energy sources such as solar, wind and hydropower. These findings build on Sarfraz et al. (2023) and Obamen et al. (2021) by suggesting that national policy initiatives, such as decreasing fossil fuel dependency and promoting renewables, are critical to achieving sustainable development. While Sarfraz et al. (2023) and Obamen et al. (2021) emphasized environmental practices, Tenaw and Beyene (2021) broadened the discussion to include the macroeconomic impacts, illustrating how environmental sustainability at the national level supports long-term economic growth. This perspective highlights the importance of a holistic approach to sustainability that incorporates both national policy and organizational practices. The critical analytical question arising from this juxtaposition is whether environmental practices influence sustainable development directly, as assumed by organisational studies, or only indirectly through other macroeconomic factors.

A regional perspective was provided by Bowmans (2022) on Kenya’s legal and policy frameworks designed to promote environmental sustainability. This study underscored Kenya’s constitutional guarantees of a clean environment, as well as its legislative initiatives, such as the Climate Change Act and the ban on plastic bags. These efforts align with the global sustainability objectives highlighted by Tenaw and Beyene (2021) and Sarfraz et al. (2023), illustrating Kenya’s proactive role in addressing climate change and environmental protection. While Bowmans (2022) emphasized the significance of regulatory frameworks and policies in driving sustainability, Sarfraz et al. (2023) and Obamen et al. (2021) focused more on environmental practices at the corporate level. Nevertheless, all three studies concur that effective environmental management, whether through policy or national initiatives, is crucial for sustainable development. Sarfraz et al. and Obamen underscore the role of CSR and EMPs at the organizational level, while Tenaw and Beyene advocate for policy-driven sustainability measures. Bowmans adds a regional dimension, highlighting how regulatory frameworks can help countries like Kenya achieve sustainability objectives. The contrast between these studies exposes a persistent gap in the literature: the lack of an integrated framework that bridges macro-level policy instruments and micro-level organisational practices. This disconnect is particularly problematic for Sub-Saharan Africa, where weak policy enforcement may render corporate environmental practices ineffective unless embedded within functioning regulatory systems.

### Theoretical framework

The study used the Ecological Modernization Theory. Theorists, such as Joseph Huber and Martin Janicke ( Mol, SpAargaren & Sonnenfeld, 2020), developed a new environmental sustainability paradigm, calling it Ecological Modernization Theory (EMT). As opposed to the classical theories on the environment, most cases of the development of industrial growth is looked at as an inevitably degrading process, EMT states that economic growth and technology can coexist and, in fact, lead to environmental sustainability. This theory suggests that changes in the regulations and structural changes in society are certain to make societies modernize with the assistance of technological innovation that offers these changes. EMT offers the formula of those industrialized nations, which may theoretically lower their ecological imprint without worsening the economic development of the state through stimulating the use of clean technologies, sustainable use of resources, and green economy policy ( Julkovski et al., 2021). This makes the theory particularly relevant in Sub-Saharan African nations, where sustainable development is of significant relevance in economic development, given the fact that the region has been reliant on natural resources and is also prone to environmental degradation.

Among the key arguments of EMT, it should be noted that technological innovation and environmental policy reforms represent the key elements of the sustainability development realization. EMT emphasizes technological change, environmental governance, and structural transformation as key drivers of ecological transition. However, due to data limitations in cross-country African panel studies, these processes are captured indirectly through environmental outcome indicators such as emissions, renewable energy use and forest cover. According to Bugden (2022), such a form of innovation provides a possibility to divide the sphere of economic activity and environmental degradation, therefore enabling countries to follow both economic and environmental goals. Moreover, EMT also emphasizes that regulatory frameworks and market-based incentives could also assist businesses and industries in utilizing sustainable practices. Such policies include the environmental taxes, the emissions trading and the subsidies on the clean technologies, which create a business case in favour of environmental responsibility, and this is the reason the clean practices should be invested in ( Xie et al., 2022). Such policies can encourage investments in green technology within the Sub-Saharan African setup that would precipitate economic growth and safeguard the environment.

EMT ontologically assumes that environmental problems are not natural problems but problems which are deeply rooted in the social and economic systems. Under this outlook, environmental concerns have a combination effect on industrial development and economic processes, and the problems should be addressed in a coordinated manner, not as individual actions ( Mol et al., 2020). EMT, in epistemological terms, is pragmatic and relies on empirical studies and policy research in order to come up with effective mechanisms that can be used to achieve a balance between the development of the economy and environmental sustainability. The theory challenges policymakers and scholars to find solutions that add to the existing economic systems as they transform into sustainable ones. This epistemological approach is applicable in the Sub-Saharan Africa scenario, where pragmatic and responsive solutions are needed because of the disparity in industrial development and environmental problems in the area.

Despite the positivity, critics argue that EMT is too optimistic about the future of the technological and regulatory solutions, and at times it underestimates the level of behavioural and cultural changes to have the actual sustainability ( Julkovski et al., 2021). Besides, the reliance of EMT on the existing technological advancement assumes the resources and infrastructures, which cannot necessarily be the case in the developing nations, including Sub-Saharan Africa. Another point that the theory has been criticized for is its focus on top-down, state-based approaches that do not give significant attention to the contribution of grassroots movements and the involvement of local communities in the process of environmental change. It is even more so in Sub-Saharan Africa, where local knowledge and community participation on most occasions is determinant of success of any environmental project ( Mol, 2001). Besides, there is the possibility that EMT will not fully offset the imbalance of power that leads to environmental exploitation since the corporations and the richer countries will have higher control over the policy-making process than the developing nations.

Based on the example of Sub-Saharan Africa, EMT introduces a valid framework for reflecting on viable aspects of the economic development plans of the region with respect to sustainable practices. The development of technology and green investments should be part of the policies that African governments adopt so that they can not only realize economic growth but also make sure that such issues as deforestation, lack of water and air pollution become a thing of the past. To illustrate the point, the renewable energy projects and green infrastructure development have already started to be embraced in such states as Kenya and South Africa, which aligns with the principles of EMT ( Salifu and Salifu, 2024). These ventures reduce the environmental effects, in addition to offering jobs and financial security. EMT therefore provides a suitable analytical lens for this study by linking technological innovation, institutional reforms, and sustainability practices to measurable development outcomes in Sub-Saharan Africa, where ecological modernization processes are still evolving and uneven across countries. Through collaboration, these stakeholders will be in a position to share resources and skills in order to develop contextual solutions to environmental sustainability. Moreover, the emphasis of EMT on policy changes and market incentives is closely connected with the interest of Sub-Saharan Africa in environmental regulations because the countries of the region strive to find a balance between economic growth and environmental sustainability.

While EMT emphasizes technological innovation and regulatory reforms as drivers of sustainability, this study operationalizes the theory by focusing on the measurable environmental outcomes that reflect successful ecological modernization. EMT’s central proposition is the decoupling of economic growth from environmental degradation. This decoupling, if occurring, should be observable through three key environmental indicators. First, greenhouse gas emissions capture the extent to which countries have reduced their carbon intensity, which is a direct outcome of EMT driven transitions to cleaner production and energy systems. Second, renewable energy consumption reflects the shift away from fossil fuels, representing the technological transformation that EMT posits as necessary for ecological modernization. Third, forest cover serves as a proxy for sustainable resource management and ecosystem preservation, indicating whether economic development has occurred alongside, rather than at the expense of, natural capital. Thus, while the study does not directly measure innovation patents or regulatory instruments, it tests the empirical consequence of EMT: that countries further along the ecological modernization pathway (lower GHG emissions, higher renewable energy use, greater forest cover) should achieve better sustainable development outcomes.

This outcome based operationalization is appropriate for the Sub-Saharan African context, where reliable cross national data on policy instruments and innovation inputs are scarce, but environmental outcomes are consistently measured by international databases such as the World Bank. Specifically, environmental practices are captured through the Environmental Practices Index (EPI), which reflects resource efficiency and ecological protection consistent with EMT’s decoupling hypothesis. The integration of this variable within a dynamic System GMM framework allows the study to empirically test EMT’s core proposition that environmental reforms and improvements in ecological efficiency influence sustainable development outcomes over time.

## Methodology

This study used a longitudinal panel design and incorporated both the descriptive and explanatory elements that looked at sustainability dynamics in the Sub-Saharan African region within the 24-year period in a comprehensive manner. The descriptive element of the design was applied to capture in a systematic manner and to document the nature, trend and current situation of the sustainability practices, institutional quality and sustainable development in the region and over time. Simultaneously, the dynamic relations and causal inferences between the independent and dependent variables were examined using the explanatory component. Precisely, the research aimed at providing an explanation of the joint effects of sustainability practices and institutional quality on sustainable development outcomes over time.

The study adopted a positivist research philosophy to investigate the relationships between sustainability practices, institutional quality and sustainable development in Sub-Saharan African countries. Grounded in the belief that reality was objective and measurable, positivism emphasized empirical evidence, hypothesis testing, and statistical analysis. The study examined data from 49 Sub-Saharan African countries over a 24-year period from 2000 to 2023 to analyze sustainability practices, institutional quality and their influence on sustainable development. The year 2000 was selected as it marked the adoption of the millennium development goals, which formally integrated environmental sustainability into the global development agenda, including SSA. In addition, the period after 2000 is a milestone in the history of the region, with a rise in attention to sustainability in the world, the adoption of the SDGs in 2015 and the development of more ESG-related policies and investment trends in the emerging economies ( United Nations, 2015; World Bank, 2023). Countries, including Eritrea, Eswatini (formerly Swaziland), Somalia and South Sudan were excluded from the final sample where consistent data across the study period (2000–2023) were not available for key variables, as incomplete time-series observations would have compromised the validity and comparability of the dynamic panel estimation.

The study relied exclusively on secondary data from reputable sources, including the World Bank Data Bank (2025), UNDP (2025), Fund for Peace (2025) and Sustainable Development Report (2024). The World Bank provided data on environmental indicators like greenhouse gas emissions, renewable energy, and forest cover. No permissions were needed as data came from open-access or licensed sources accessed via institutional subscriptions. The Environmental Practices Index (EPI) was constructed as a composite indicator to capture the multidimensional nature of environmental sustainability across Sub-Saharan African countries. The EPI comprised three component indicators sourced from the World Bank Data Bank (2025): GHC emissions, renewable energy and forest area cover. Each component was first normalized using min max scaling to a 0 to 1 scale to ensure comparability across countries and over time, given differences in measurement units. For GHC emissions, where higher raw values represent poorer environmental outcomes, an inverse transformation was applied by subtracting the normalized value from 1, ensuring that higher EPI scores consistently reflect better environmental performance. Renewable energy and forest area cover, where higher values reflect better outcomes, were used without inverse transformation. The three transformed and normalized indicators were then aggregated using a linear weighting approach, where equal weights were assigned to each component to avoid subjective bias in index construction. This assumes substitutability among components, a standard practice in composite indices justified by their shared environmental policy objective. The resulting EPI provides a standardized measure of environmental practices suitable for panel regression analysis and cross country comparison over the 24 year study period.

After data cleaning, descriptive statistics, that is, standard deviations and means, were conducted for each variable. Data analysis encompassed the use of descriptive statistics and regression models. Stata Version 16 was employed for the analysis, ensuring sophisticated analysis and interpretation of the data. The data was then presented in tables. To address potential endogeneity, unobserved heterogeneity, and dynamic panel bias, the study employed the two-step System Generalized Method of Moments (System GMM). To minimize the risk of instrument proliferation and model overfitting, the instrument count was restricted relative to the number of cross-sectional units by limiting lag depth and applying the collapse option during estimation. Instrument validity and exogeneity were assessed using the Hansen J-test and Difference-in-Hansen tests, while the Arellano-Bond serial correlation tests were used to confirm the absence of second-order autocorrelation in the differenced residuals. The resulting GMM model equation, as by Blundell and Bond (1998) are below;

$$Y_{\mathrm{it}}=\beta_{0}+\beta_{1}Y_{1\mathrm{it}-1}+\sum_{k=1}^{K}\beta_{2k}{\mathrm{ENV}}_{k,\mathrm{it}}+\mu_{\mathrm{it}}+\epsilon_{\mathrm{it}}$$ (3.1a)

$$Y_{\mathrm{it}}=\beta_{0}+\beta_{1}Y_{1\mathrm{it}-1}+\sum_{k=1}^{K}\beta_{2k}{\mathrm{ENV}}_{k,\mathrm{it}}+{\mathrm{CV}}_{\mathrm{it}}+\mu_{\mathrm{it}}+\epsilon_{\mathrm{it}}$$ (3.1b)

Where;

$Y_{\mathrm{it}}\;$ = Dependent Variables over time period.

$\;Y_{1\mathrm{it}-1}$ = One time period Lag of the dependent variables.

$\sum_{k=1}^{K}F_{k,\mathrm{it}}\;$ = Is a set of one Independent variable indicator.

${\mathrm{INV}}_{k,\mathrm{it}},{\mathrm{SOC}}_{k,\mathrm{it}},{\mathrm{GOV}}_{k,\mathrm{it}}\;\text{and}\;{\mathrm{ECO}}_{k,\mathrm{it}}$ = Independent variables over time period.

$\beta_{0}$ = Intercept.

$\beta_{1}-\beta_{2}\;$ = Slope of Coefficients.

${\mathrm{CV}}_{\mathrm{it}}$ = Control variables over time period.

$\mu_{\mathrm{it}}$ = Country-specific effects over time period.

$\epsilon_{\mathrm{it}}$ = Error term over time period.

## Results and discussion

### General information

The study utilized secondary data from 49 Sub-Saharan African countries covering the period 2000–2023. Data represented a balanced panel dataset, with annual observations across the 24-year period. Initially, the dataset consisted of 1,176 observations based on 49 SSA countries, but was reduced to 1,080 as Eritrea, Eswatini (formerly Swaziland), Somalia and South Sudan were deleted due to missing data. No data were available for these four countries in the World Bank database, resulting in their exclusion from the final sample. All indicators were measured annually and standardized to indices to ensure comparability across countries and over time. The dataset captured environmental practices sustainable development outcomes. Table 1 presents a summary of the dataset characteristics.

**Table 1.** General information on study data.

Indicator	Description	Coverage
Countries Included	Sub-Saharan African countries	45
Study Period	Time frame of data collected	24 years (2000–2023)
Unit of Analysis	Country-level annual observations	Panel data
Number of Observations	Total dataset size	1,080 (45 × 24 years)
Panel Structure	Dataset structure	Strongly balanced panel
Measurement Periodicity	Frequency of data collection	Annual
Data Type	Nature of data used	Secondary quantitative and qualitative document analysis

### Descriptive statistics

This section presents the descriptive statistics of the study variables, including environmental practices and sustainable development, for the 45 Sub-Saharan African countries over the period 2000–2023. The results summarize the distribution of the variables in terms of their mean and standard deviation, providing an overview of central tendencies and variations across countries and years.

The descriptive statistics for environmental indicators showed that the GHG Emissions Index had the highest mean (M = 0.894, SD = 0.148), reflecting generally favourable emissions performance across SSA, with relatively low variation among countries. The Renewable Energy Consumption Index recorded a moderate average (M = 0.644, SD = 0.268), suggesting uneven adoption of renewable energy across the region. The Forest Area Cover Index posted the lowest mean (M = 0.326, SD = 0.252), indicating limited forest coverage with substantial variation among countries. These results highlight that while emissions levels are relatively low, renewable energy adoption is moderate, and forest conservation remains a significant regional challenge, underscoring persistent environmental sustainability concerns.

The descriptive statistics for sustainable development measures showed mixed progress with positive environmental adjustments. The Human Development Index recorded a moderate average (M = 0.517, SD = 0.112), reflecting ongoing development challenges. The Adjusted Net Savings Index showed reasonable performance (M = 0.571, SD = 0.128). The Child Mortality Index reflected substantial health improvements (M = 0.840, SD = 0.096), though with remaining disparities between countries. These results present a portrait of moderate development progress that is constrained by persistent structural challenges ( Table 2).

**Table 2.** Descriptive statistics for the variables.

Variable	Obs	Mean	Std. Dev.
ghg_index	1,080	0.894	0.148
ren_en_index	1,080	0.644	0.268
forest_cov_index	1,080	0.326	0.252
hdi_index	1,080	0.517	0.112
nat_save_index	1,080	0.571	0.128
mort_ind_index	1,080	0.840	0.096

Note: All indices normalized to 0–1 scale, where higher values indicate better performance.

The observed pattern of relatively strong emissions performance coupled with forest conservation challenges reflects documented regional characteristics. Sub-Saharan Africa’s per capita CO _2_ emissions are low ( World Bank, 2023). Conversely, the FAO (2020) reports the region accounted for the highest net loss of forest area of any region between 2010 and 2020, identifying it as a primary deforestation hotspot.

### Panel unit root tests and stationarity tests

The study used both the Levin-Lin-Chu (LLC) test and the Fisher-type Augmented Dickey-Fuller (ADF) and Phillips-Perrson (PP) tests to assess stationarity. The first test, LLC, was originally performed as a preliminary test in the context of common unit root processes across panels. But this assumption is sometimes restrictive and might not be true for heterogeneous panel data sets, so Fisher-type ADF and PP tests were used as the basis for deciding stationarity of the panel data sets. A variable was considered stationary only when both the ADF and PP tests did not accept the null hypothesis of no unit root at the 5% level.

Based on LLC tests, environmental indicators such as the GHG Emissions Index (ghg_index) and the Renewable Energy Consumption Index (ren_en_index) were strongly stationary. The results for the variables confirm they meet the critical assumption of stationarity required for subsequent panel regression analysis ( Table 3).

**Table 3.** Levin-Lin-Chu unit-root test for the variables.

Variable	Unadjusted t	Adjusted t*	p-value	Stationarity
ghg_index	−15.7913	−5.2572	0.0000	Stationary
ren_en_index	−14.1928	−5.6037	0.0000	Stationary
forest_cov_index	−8.5448	−3.4510	0.0003	Stationary
hdi_index	−9.4484	−2.4561	0.0070	Stationary
nat_save_index	−8.1004	−8.1004	0.0000	Stationary
mort_ind_index	−34.3783	−30.6473	0.0000	Stationary

Note: H _0_: Panel contains unit roots (non-stationary).

The Fisher-type panel unit root test results, presented in Table 4, indicate that GHG Emissions Index (ghg_index) demonstrate strong evidence of stationarity. The Renewable Energy Consumption Index (ren_en_index) and Forest Area Cover Index (forest_cov_index) show evidence of non-stationarity, with both tests failing to reject the null hypothesis (p > 0.05). The findings are shown in Table 4.

**Table 4.** Unit root test using ADF and PP (Fisher type) tests.

Variable	ADF test	p-value	PP test	p-value	Stationarity
ghg_index	2.6786	0.0037	2.8060	0.0025	Stationary
ren_en_index	0.0162	0.4935	−0.9599	0.8314	Non-stationary
forest_cov_index	−2.5829	0.9951	−3.1866	0.9993	Non-stationary
hdi_index	−1.0158	0.8451	−1.4473	0.9261	Non-stationary
nat_save_index	5.2035	0.0000	18.8535	0.0000	Stationary
mort_ind_index	38.8301	0.0000	17.6178	0.0000	Stationary

Note: H _0_: Panel contains unit roots (non-stationary).

The results of the Fisher-type panel unit root tests on the first-differenced series, presented in Table 5, demonstrate that the transformation successfully achieved stationarity for both previously non-stationary variables. For each differenced series, both the Augmented Dickey-Fuller (ADF) and Phillips-Perron (PP) tests decisively reject the null hypothesis of non-stationarity at the 1% significance level (p = 0.0000).

**Table 5.** First differencing using ADF and PP (Fisher type) tests.

Variable	ADF test	p-value	PP test	p-value	Stationarity
d_ren_en_index	40.6475	0.0000	182.7248	0.0000	Stationary
d_forest_cov_index	42.4784	0.0000	210.7608	0.0000	Stationary
d_hdi_index	30.0216	0.0000	211.6077	0.0000	Stationary

Note: H _0_: Panel contains unit roots (non-stationary).

The exceptionally large test statistics across all variables provide strong evidence of stationarity. The Forest Area Cover Index (d_forest_cov_index) exhibited the most substantial evidence of stationarity in the PP tests, with statistics of 210.76. Similarly, the Renewable Energy Consumption Index (d_ren_en_index) showed robust stationarity with an ADF statistic of 40.65 and a PP statistic of 182.72.

These results confirm that the first-difference transformation effectively eliminated the unit roots present in the original level series. Consequently, all two differenced variables, including d_ren_en_index and d_forest_cov_index, are now integrated of order zero, I(0) and meet the stationarity assumption required for valid panel regression analysis. The transformation enables their inclusion alongside the nine originally stationary variables in subsequent econometric modelling without spurious regression concerns.

### Multicollinearity test

The Variance Inflation Factor (VIF) analysis confirms the absence of harmful multicollinearity among all independent variables across the different model specifications. For every variable group, both individual and mean VIF values are substantially below the standard critical threshold of 10, with most falling well below the more conservative threshold of 5. Environmental practices demonstrate minimal multicollinearity with a mean VIF of 1.03 and individual values of 1.02–1.04. These results provide strong evidence that the explanatory variables are not excessively correlated, ensuring that the parameter estimates in subsequent regression analysis will be stable, reliable, and interpretable. Therefore, the dataset is robust and suitable for multivariate panel estimation without multicollinearity concerns ( Table 6).

**Table 6.** Multicollinearity test results.

DV	Independent variables	VIF	1/VIF	Mean VIF
SD	ghg_index, d_ren_en_index, d_forest_cov_index	1.02, 1.03, 1.04	0.978, 0.969, 0.958	1.03

### System GMM model diagnostics

The system GMM regression results reveal significant insights into the determinants of sustainable development (SD) across Sub-Saharan Africa. The lagged dependent variable (L.SD) demonstrates a strong positive and statistically significant coefficient (β = 0.593, p < 0.001), indicating substantial persistence in sustainable development outcomes. This suggests that previous levels of sustainable development strongly influence current performance, with approximately 59% of prior SD levels carrying forward to the current period. This finding underscores the path-dependent nature of sustainable development, where past achievements create momentum for future progress.

Among environmental practices, none of the variables achieved statistical significance at conventional levels. The GHG Emissions Index (β = 0.103, p = 0.141), Renewable Energy Consumption Index (β = 0.047, p = 0.672), and Forest Area Cover Index (β = −6.466, p = 0.163) all showed statistically insignificant relationships with sustainable development. This suggests that, in the comprehensive model accounting for all other factors, individual environmental indicators may not directly drive sustainable development improvements or their effects may be mediated through other channels ( Table 7).

**Table 7.** Two-Step system GMM estimation results.

Variable	Coefficient (β)	Std. Error	t-statistic	p-value	95% Confidence Interval
**Lagged Dependent Variable**					
L.SD	0.5933	0.0946	6.27	0.000***	(0.4078, 0.7789)
**Environmental Practices**					
ghg_index	0.1030	0.0700	1.47	0.141	(−0.0342, 0.2403)
d_ren_en_index	0.0468	0.1104	0.42	0.672	(−0.1697, 0.2633)
d_forest_cov_index	−6.4661	4.6393	−1.39	0.163	(−15.5589, 2.6266)

The model diagnostics confirm the validity of the estimation approach. The Arellano-Bond tests show appropriate serial correlation patterns, with significant AR (1) (p = 0.007) and insignificant AR (2) (p = 0.798), satisfying the key assumption for GMM estimation ( Table 8).

**Table 8.** Arellano-Bond test for serial correlation.

Test	z-statistic	p-value	Conclusion
AR (1) in first differences	−2.69	0.007	Serial correlation present
AR (2) in first differences	0.26	0.798	No serial correlation

The Hansen J-test of overidentifying restrictions showed a χ ^2^ statistic of 28.54 with 22 degrees of freedom and a p-value of 0.156, failing to reject the null hypothesis that the instruments are valid. This non-significant result confirms the validity of the instrument set used in the system GMM estimation. The Difference-in-Hansen test for the GMM instruments for levels yielded a χ ^2^ statistic of 8.67 with 10 degrees of freedom and a p-value of 0.564, indicating that the exogeneity assumption for this instrument subset cannot be rejected. To minimize the risk of instrument proliferation, the instrument count was restricted to 38, which is below the number of cross sectional units (45 countries), by limiting lag depth to a maximum of two lags for the dependent variable and endogenous predictors and applying the collapse option. These diagnostics confirm the appropriateness of the model specification and the reliability of the instruments used ( Table 9).

**Table 9.** Instrument validity by Hansen J-test and Difference-in-Hansen test.

Test	χ ^2^ statistic	df	p-value	Conclusion
Hansen J-test of overid. Restrictions	24.49	974	1.000	Instruments valid
Difference-in-Hansen (GMM instruments for levels)	−7.83	94	1.000	Exogeneity not rejected

The White’s test was conducted to test the null hypothesis (H _0_) that the regression model exhibits homoskedasticity (constant variance of the error term). The results provide strong evidence to reject the null hypothesis of homoskedasticity at the 1% significance level. This conclusion is based on the statistically significant chi-squared statistic for the heteroskedasticity component (χ ^2^ (135) = 268.70, p < 0.001).

The decomposition of the IM-test reveals distinct patterns in the error structure. While the heteroskedasticity component is highly significant (p < 0.001), the skewness component is not statistically significant at conventional levels (χ ^2^ (15) = 15.12, p = 0.443), indicating that the distribution of residuals is approximately symmetric. However, the kurtosis component shows statistical significance (χ ^2^ (1) = 4.31, p = 0.038), suggesting some deviation from normal distribution in the tails of the residual distribution. The combined total test statistic (χ ^2^ (151) = 288.12, p < 0.001) confirms the overall presence of non-spherical errors. These results indicate that while the residuals are symmetrically distributed, they exhibit heteroskedasticity and some non-normal kurtosis. This finding validates the use of robust standard errors in the GMM estimation to address the heteroskedasticity concern and ensure reliable inference despite the non-normal error distribution characteristics ( Table 10). To address the heteroskedasticity identified, the study employed two-step system GMM estimation with Windmeijer-corrected standard errors, which are robust to heteroskedasticity and provide more reliable inference in the presence of non-spherical errors.

**Table 10.** White’s Test for Heteroskedasticity.

Test component	Chi ^2^	df	P-value
Heteroskedasticity	268.70	135	0.0000
Skewness	15.12	15	0.4430
Kurtosis	4.31	1	0.0380
Total	288.12	151	0.0000

Before model estimation, a comprehensive series of diagnostic tests was conducted to ensure the robustness and validity of the empirical strategy. Panel unit root tests confirmed the stationarity of the transformed series. Tests for multicollinearity indicated that the explanatory variables were not highly correlated, mitigating concerns of inflated variances. Critically, the specification of the dynamic panel model was validated: the Arellano-Bond test confirmed the absence of second-order serial correlation in the differenced errors, and the Hansen J-test supported the validity of the instrument set. With all key econometric assumptions satisfied, the study proceeded to estimate the System GMM models to test the formulated hypotheses and draw causal inferences.

### System GMM model

The aggregated EPI was used in the main hypothesis test to capture the combined environmental effect, rather than testing each component separately. Although the individual components were not significant in isolation ( Table 7), this does not undermine the index approach, as composite indices are designed to reflect multidimensional interactions that may not be detectable in single-component regressions. The EPI used in Table 11 was constructed from the differenced stationary components of the three indicators, ensuring consistency in stationarity treatment across both Table 7 and Table 11 specifications. In this regard, the system GMM results for the hypothesis indicate that the Environmental Practices Index (EPI) has a statistically insignificant relationship with sustainable development (β = −0.0329, p = 0.624). The lagged dependent variable shows strong persistence (β = 0.8124, p < 0.001), explaining approximately 81% of current sustainable development levels, as shown in Table 11.

**Table 11.** Environmental practices and sustainable development.

Variable	Coefficient (β)	Std. Error	z-statistic	p-value	95% Confidence Interval
**Lagged Dependent Variable**					
L.SDI	0.8124	0.0218	37.27	0.000***	(0.7697, 0.8552)
EPI	−0.0329	0.0670	−0.49	0.624	(−0.1643, 0.0985)
_cons	0.1019	0.0208	4.90	0.000***	(0.0611, 0.1428)

H _01_: Environmental practices do not influence the sustainable development of the Sub-Saharan African countries.

This finding suggests that, contrary to the theoretical expectations established in the literature review ( Hoshino & Seixas, 2019; Shahzad et al., 2020; Qalati et al., 2023), current environmental sustainability measures in Sub-Saharan Africa do not translate into measurable improvements in composite sustainable development outcomes. The negative, albeit insignificant, coefficient even hints at potential misalignment or inefficiencies in how environmental policies are implemented or integrated within broader development frameworks in the region. This empirical result provides a critical counterpoint to studies like Obamen et al. (2021) and Sarfraz et al. (2023), which reported positive relationships, suggesting that the institutional, economic, or implementation context in SSA may disrupt the theoretical link between environmental stewardship and holistic development.

This divergence may be attributed to fundamental differences in institutional quality and governance structures between SSA and the contexts examined in those studies. Unlike the manufacturing sector in Nigeria or the rapidly industrializing economy of China, which benefit from stronger regulatory enforcement and more cohesive policy implementation, SSA countries grapple with pervasive corruption, weak monitoring systems and competing developmental priorities that often relegate environmental concerns to the periphery. These contextual factors likely undermine the effectiveness of environmental policies, explaining why the positive relationships observed elsewhere do not materialize in the SSA setting.

The study finds that environmental practices do not have a statistically significant influence on sustainable development in Sub-Saharan Africa. From the perspective of EMT, this result suggests that the region is still at an early or incomplete stage of ecological modernization, where environmental reforms have not yet been fully integrated into economic and institutional systems to generate transformative sustainability outcomes. Although many Sub-Saharan African countries have established formal environmental policies and regulatory frameworks, their implementation remains fragmented and inconsistent due to weak institutional capacity, poor governance structures, limited financial resources, and inadequate technical expertise. In addition, political instability and competing development priorities often divert attention and resources away from environmental sustainability agendas. Weak monitoring and enforcement mechanisms further constrain compliance, limiting the effectiveness of environmental interventions. As a result, environmental practices have not yet reached the scale, coherence, and institutional embedding required under EMT to produce measurable impacts on broader sustainable development outcomes.

These findings raise questions about the applicability of EMT to the Sub-Saharan African context, where the institutional prerequisites for ecological modernization are often lacking. This aligns with critiques that EMT assumes institutional capacity that may not exist in peripheral regions, suggesting that alternative theoretical frameworks such as political ecology or world-systems theory may be more appropriate for understanding the environment-development nexus in SSA. Political ecology may attribute the null result to unequal power relations and resource access, where environmental policies are shaped by elite interests rather than genuine sustainability goals. World-systems theory, on the other hand, may locate the failure within the global economic structure, where peripheral regions remain locked into resource extraction and dependency relationships that undermine local environmental governance.

The lagged dependent variable shows strong and highly significant persistence, explaining significant variation in current sustainable development levels. This overwhelming path dependence is a crucial finding. It indicates that sustainable development in SSA is predominantly determined by its own historical trajectory, creating a high degree of inertia. This result aligns with Martin and Dahlstrom’s (2020) view that the benefits of environmental sustainability may not be immediate and could be overshadowed by entrenched structural factors. The strong autoregressive effect suggests that breaking from historical development paths to incorporate new sustainability paradigms is exceptionally challenging, potentially explaining why current environmental practices fail to show a significant independent impact.

This empirical outcome necessitates a critical re-evaluation of the policy frameworks discussed by Bowmans (2022) and the implementation mechanisms highlighted by Agweny (2021). While countries like Kenya have established progressive environmental legislation, such as the Climate Change Act and plastic bag bans, and sectors like freight transport show cost benefits from green practices, these isolated successes do not aggregate to a significant macroeconomic relationship at the regional level. The findings suggest that environmental practices in SSA have not yet reached the scale or institutional embedding required to produce measurable impacts on broader sustainable development outcomes. This supports Ying’s (2022) sector-specific findings, suggesting that environmental benefits may be context-dependent and not automatically translate to national or regional development metrics.

The Arellano-Bond test results for the Environmental Practices model show appropriate serial correlation patterns for valid GMM estimation. The significant AR (1) test (z = −2.52, p = 0.012) indicates first-order serial correlation in the first-differenced errors, which is expected and acceptable in dynamic panel models. The insignificant AR (2) test (z = 1.09, p = 0.275) confirms the absence of second-order serial correlation, satisfying the key assumption that the error term in the level equation is not serially correlated. Through restricting lag depth to a maximum of two lags for the dependent variable and endogenous predictors and using the collapse option, the instrument count was limited to 38, which is less than the number of cross-sectional units (45 countries), in order to reduce the danger of instrument proliferation. This pattern validates the moment conditions and supports the reliability of the GMM estimator ( Table 12).

**Table 12.** Arellano-Bond test for serial correlation for environmental practices model.

Test	z-statistic	p-value	Conclusion
AR(1) in first differences	−2.52	0.012	Serial correlation present
AR(2) in first differences	1.09	0.275	No serial correlation

The instrument validity tests confirm the appropriateness of the instrument set used in the Environmental Practices GMM estimation after addressing concerns regarding instrument proliferation. To minimize the risk of overfitting, the instrument count was restricted to 38, which is below the number of cross sectional units (45 countries), by limiting lag depth to two lags for the dependent variable and endogenous predictors and applying the collapse option. The Hansen J-test yields a χ ^2^ statistic of 19.89 with 22 degrees of freedom and a p-value of 0.101, failing to reject the null hypothesis that the instruments are valid. The Difference-in-Hansen test for the GMM instruments for levels shows a χ ^2^ statistic of 6.77 with 10 degrees of freedom and a p-value of 0.441, indicating that the exogeneity assumption for this instrument subset cannot be rejected. These results collectively support the validity of the instruments ( Table 13).

**Table 13.** Instrument validity tests for environmental practices model.

Test	χ ^2^ statistic	df	p-value	Conclusion
Hansen J-test of overid. restrictions	19.89	22	0.101	Instruments valid
Difference-in-Hansen (GMM instruments for levels)	6.77	10	0.441	Exogeneity not rejected

## Conclusion

The study concludes that current environmental sustainability measures in Sub-Saharan Africa do not significantly influence sustainable development outcomes. The statistically insignificant relationship between the Environmental Practices Index and sustainable development suggests that environmental policies and initiatives in the region may not be effectively designed, implemented or integrated to significantly improve sustainable development outcomes.

Existing environmental policies require substantive enhancement through better integration and implementation mechanisms. The insignificant relationship between environmental practices and sustainable development suggests that current policies may be inadequately designed or poorly enforced. Policymakers should develop integrated environmental sustainability frameworks that explicitly link specific environmental interventions such as emissions control, renewable energy adoption and forest conservation to measurable sustainable development outcomes, with clear monitoring and evaluation systems to track progress and impact. Specific instruments such as carbon taxes, renewable energy subsidies, or payments for forest conservation could be considered, though their effectiveness would depend on strengthening the institutional capacity required for implementation and enforcement in SSA.

### Limitations

This study acknowledges several limitations that may affect the interpretation and generalizability of its findings. First, the research relied on secondary data from World Bank, which may contain measurement errors, inconsistencies in data collection methodologies across countries, and missing values that required imputation. While these sources represent the most comprehensive data available for cross-country analysis in Sub-Saharan Africa, variations in national statistical capacities and reporting standards may introduce measurement biases. To mitigate this limitation, the study employed rigorous data cleaning procedures, consistency checks, and standardized normalization techniques across all variables.

The study period of 24 years may be insufficient to capture lagged effects, as environmental practices often require longer time horizons to translate into measurable sustainability outcomes. Threshold effects may also exist, whereby environmental investments must reach a minimum scale before producing detectable impacts, a possibility not examined in this analysis. The study did not explore potential moderating or mediating factors such as institutional quality, governance, or financial development, which could influence the relationship between environmental practices and sustainable development. Reverse causality remains plausible as well, since sustainable development may enable environmental practices rather than the reverse. Further, the analysis did not include additional robustness checks such as alternative estimators (pooled OLS, fixed effects, difference GMM), sub-period analysis (pre- versus post-2015 SDG adoption), exclusion of outliers (e.g., Mauritius), alternative lag structures, or alternative dependent variables. These alternative pathways should be investigated in future research.

## Ethics and consent

Ethical approval and consent were not required.

## Data Availability

https://www.stata.com/stata16/. All analyses were conducted using STATA Version 16.
