# Supplementary material for: Influence of Environmental Practices on Sustainable Development of the Sub-Saharan African Countries
Source: Open Res Afr. 2026 Jul 7;9:10. Originally published 2026 Mar 25. [Version 2] doi: 10.12688/openresafrica.16443.2 (PMC13550923; doi:10.12688/openresafrica.16443.2)
Supplement: Peer review history

# Reviewer report: Reviewer response for version 2

**DOI:** 10.21956/openresafrica.17980.r35218

**Published:** 2026-07-15

**Authors:** Mónica Brito

**Affiliations:** 1 University of Évora, Évora, Portugal

The revised version of the manuscript demonstrates a significant effort on the part of the authors to address the comments made during the peer-review process. Overall, the changes made have enhanced the scientific quality of the work, improving the theoretical framework, the methodological description, the presentation of the results and the discussion of their implications.

 Of particular note is, firstly, the inclusion of a section dedicated to identifying the research gap and clarifying the study’s contribution, which provides a clearer understanding of the originality of the research and its contribution to the literature on sustainable development in sub-Saharan Africa. The methodological framework has also been strengthened through a more detailed description of the construction of the Environmental Practices Index, making the procedure for constructing the indicator more transparent and reproducible.

 The link between Ecological Modernisation Theory and the empirical variables is now more consistent, with the conceptual framework justifying the use of the selected environmental indicators being made explicit. The literature review also demonstrates greater critical analysis, establishing links between different studies and better contextualising the results obtained.

 One of the most positive aspects of this version is the more in-depth discussion of the empirical results. Rather than interpreting the statistically non-significant effect as a lack of scientific relevance, the authors discuss possible institutional, structural and contextual explanations that help to understand the complexity of the relationship between environmental practices and sustainable development in the region studied. This approach lends greater scientific maturity to the manuscript and demonstrates a prudent interpretation of the results.

 The inclusion of a specific section on the study’s limitations also constitutes a significant improvement, as it transparently acknowledges the constraints associated with the data used, the methodological choices and the scope for future research.

 Despite these improvements, some minor inconsistencies remain that warrant attention prior to indexing. In particular, it is recommended that the information regarding the final number of countries included in the sample be standardised, ensuring consistency between the abstract, the methodology and the other sections of the manuscript. It is also suggested that the consistency of the results reported for the diagnostic tests of the System GMM model be checked, ensuring that all tables and descriptions correspond to the final version of the estimation. These issues appear to stem primarily from matters of editing and text harmonisation, and do not call into question the robustness of the methodology or the study’s conclusions.

 Similarly, a final linguistic review is recommended to eliminate minor grammatical errors and standardise the terminology used throughout the manuscript, thereby contributing to a more fluid and consistent presentation.

 In summary, I consider that the authors have responded appropriately to the comments from the previous review and that the manuscript represents a substantial improvement on the version initially submitted. The remaining issues are of a minor and editorial nature, and do not affect the scientific quality of the work, the validity of the methodology employed, or the relevance of the conclusions presented.

Is the work clearly and accurately presented and does it cite the current literature?

Partly

If applicable, is the statistical analysis and its interpretation appropriate?

I cannot comment. A qualified statistician is required.

Are all the source data underlying the results available to ensure full reproducibility?

Partly

Is the study design appropriate and is the work technically sound?

Partly

Are the conclusions drawn adequately supported by the results?

Partly

Are sufficient details of methods and analysis provided to allow replication by others?

No

Reviewer Expertise:

Sustainable Development; Tourism; Tourism planning and development

I confirm that I have read this submission and believe that I have an appropriate level of expertise to confirm that it is of an acceptable scientific standard, however I have significant reservations, as outlined above.

# Reviewer report: Reviewer response for version 2

**DOI:** 10.21956/openresafrica.17980.r35220

**Published:** 2026-07-09

**Authors:** Agnes Mutiso

**Affiliations:** 1 Kirinyaga University, Kirinyaga County, Kenya

I confirm that the comments that I raised for the article  "Influence of Environmental Practices on Sustainable Development of the Sub-Saharan African Countries" have been adequately addressed.

Is the work clearly and accurately presented and does it cite the current literature?

Yes

If applicable, is the statistical analysis and its interpretation appropriate?

Yes

Are all the source data underlying the results available to ensure full reproducibility?

Yes

Is the study design appropriate and is the work technically sound?

Yes

Are the conclusions drawn adequately supported by the results?

Partly

Are sufficient details of methods and analysis provided to allow replication by others?

Yes

Reviewer Expertise:

I am a senior Researcher in Corporate governance and development

I confirm that I have read this submission and believe that I have an appropriate level of expertise to confirm that it is of an acceptable scientific standard.

# Reviewer report: Reviewer response for version 1

**DOI:** 10.21956/openresafrica.17630.r34276

**Published:** 2026-06-06

**Authors:** Saqib Mehmood

**Affiliations:** 1 Xi’an Fanyi University, Xi’an, China

**1. Critical Flaws in System GMM Model Specification and Diagnostics**

******1.1. Hansen J-test p-value = 1.000 (Suspicious)******

 The Hansen test of overidentifying restrictions yields a p-value of exactly 1.000 (Page 11, Table 9; Page 13, Table 13).

 This is highly unusual in applied econometrics and typically indicates instrument proliferation — too many instruments relative to the number of cross-sectional units (45 countries).

 A p-value of 1.000 suggests the test has no power to detect invalidity, not that instruments are perfectly valid.

******1.2. Negative Chi-squared in Difference-in-Hansen Test******

 The Difference-in-Hansen test reports negative χ² statistics: -7.83 (Page 11) and -0.90 (Page 13).

 A chi-squared statistic cannot be negative. This is a technical impossibility and suggests:

 Model misspecification

 Software coding error

 Improper manual calculation or reporting

 This error alone undermines confidence in all GMM results.

******1.3. Number of Instruments Not Reported******

 The paper fails to report the total number of instruments used in the GMM estimation.

 A standard diagnostic requires that the number of instruments should be less than or equal to the number of countries (45). Without this information, readers cannot assess instrument proliferation.

******1.4. Inappropriate AR(2) Interpretation******

 The Arellano-Bond test for AR(2) yields p = 0.275 and p = 0.798 (Pages 11–12).

 While insignificant AR(2) is required, the authors do not report the number of lags used or justify why higher-order autocorrelation was not tested.

**2. Opaque Construction of the Environmental Practices Index (EPI)**

******2.1. No Definition or Formula Provided******

 The EPI is introduced as a key independent variable in Table 11, but the paper never explains how it is constructed.

 It is unclear whether EPI is:

 A simple average of GHG emissions, renewable energy, and forest cover indices

 A weighted index

 A factor score from principal component analysis

******2.2. Unit Mismatch in Components******

 The three environmental components are measured on different scales (even after normalization), but no justification is given for aggregating them.

 GHG emissions (global pollutant), renewable energy (production/consumption metric), and forest cover (stock variable) are conceptually distinct. Aggregating them into a single index assumes substitutability without justification.

******2.3. No Robustness Check with Individual Components******

 The main hypothesis test uses the aggregated EPI. However, Table 7 shows that none of the three individual components are statistically significant either.

 The authors do not explain why they switched from individual components (Table 7) to an index (Table 11) or discuss whether results are consistent across specifications.

**3. Misalignment Between Theoretical Framework and Empirical Findings**

******3.1. Theory Predicts Positive Relationship; Study Finds Insignificant Negative Relationship******

 The study explicitly adopts Ecological Modernization Theory (EMT) , which posits that technological innovation and environmental policies can decouple economic growth from environmental degradation.

 EMT predicts a positive, significant relationship between environmental practices and sustainable development.

 The study finds β = -0.0329, p = 0.624 (negative and insignificant).

******3.2. No Critical Engagement with Theory Failure******

 The discussion does not:

 Question the applicability of EMT to Sub-Saharan Africa

 Propose alternative theoretical frameworks (e.g., Political Ecology, World-Systems Theory, Post-Development Theory)

 Acknowledge that EMT has been criticized for assuming institutional capacity that may not exist in SSA (already noted on Page 6 but never connected to the null result)

******3.3. Contradiction Acknowledged but Not Explained******

 On Page 12, the authors note that results "provide a critical counterpoint" to studies like Obamen et al. (2021) and Sarfraz et al. (2023).

 However, they do not explain why SSA might differ from those contexts (e.g., China, Nigeria manufacturing sector). Possible explanations (weak enforcement, corruption, poor data quality) are mentioned but not tested or elaborated.

**4. Weak Discussion of Null Findings**

******4.1. Superficial Explanation******

 The discussion of the non-significant result (Page 12) is limited to:

 "Environmental practices may be too fragmented"

 "Inadequately scaled"

 "Insufficiently integrated"

 These are assertions, not conclusions drawn from evidence. No data or diagnostic tests support these claims.

******4.2. No Exploration of Alternative Explanations******

 The paper does not consider or test alternative explanations such as:

 Measurement error in environmental or sustainable development indicators

 Lag effects (environmental practices may take >24 years to show impact)

 Threshold effects (minimum scale of environmental investment required)

 Moderation or mediation (e.g., institutional quality, governance, financial development)

 Reverse causality (sustainable development enabling environmental practices, not vice versa)

******4.3. No Comparison with Conflicting Literature******

 The literature review cites multiple studies finding positive relationships (Hoshino & Seixas, 2019; Shahzad et al., 2020; Qalati et al., 2023; Obamen et al., 2021; Sarfraz et al., 2023).

 The discussion does not systematically compare sample characteristics, methods, or contexts to explain why this study differs.

**5. Methodological and Statistical Problems**

******5.1. Inconsistent Unit Root Test Results******

 Table 4 (Fisher-type tests) shows that renewable energy index (p = 0.4935 for ADF; p = 0.8314 for PP) and forest cover index (p = 0.9951 for ADF; p = 0.9993 for PP) are non-stationary at levels.

 The authors correctly difference these variables (Table 5).

 However, in Table 7, they use d_ren_en_index and d_forest_cov_index (differenced), but in Table 11, they use an aggregated EPI without clarifying whether EPI includes differenced or level components. This is inconsistent.

******5.2. Heteroskedasticity Confirmed but Under-discussed******

 White's test (Table 10) shows significant heteroskedasticity (χ² = 268.70, p < 0.001).

 The authors state that "robust standard errors" were used, but they do not specify which type (Huber-White, clustered, or two-step corrected).

 In system GMM with heteroskedasticity, two-step standard errors with Windmeijer correction are recommended. The paper does not confirm this.

******5.3. No Sensitivity or Robustness Checks******

 The following standard robustness checks are absent:

 Alternative estimators (e.g., pooled OLS, fixed effects, difference GMM)

 Sub-period analysis (pre- vs. post-2015 SDG adoption)

 Exclusion of outliers (e.g., Mauritius, which ranks much higher than other SSA countries)

 Alternative lag structures (e.g., 2-year, 3-year lags)

 Alternative dependent variables (e.g., separate HDI, adjusted net savings, child mortality instead of composite)

******5.4. Sample Reduction Not Justified******

 Four countries (Eritrea, Eswatini, Somalia, South Sudan) were deleted due to missing data, reducing the sample from 49 to 45 countries.

 No analysis is provided on whether missing data are random or systematic. If poorer or more fragile countries are missing, results may be biased toward more stable countries.

**6. Unclear Research Gap and Contribution**

******6.1. Stated Gap Is Not Convincing******

 The paper claims "limited research on adoption of sustainable development" in SSA (Page 1, Page 4).

 However, the literature review cites multiple recent studies on exactly this topic: Ogede et al. (2024), Tenaw & Beyene (2021), Uduji et al. (2019), Sarkodie & Adams (2020), Ojeyinka & Osinubi (2022), Sun et al. (2020).

 The paper does not explain what specific gap remains after these studies.

******6.2. No Methodological Contribution Claimed******

 The paper uses system GMM, which is advanced but not novel for this research question.

 No comparison is made with previous methods (e.g., earlier studies used panel OLS, fixed effects, or cross-sectional regressions), so the advantage of GMM is not demonstrated.

******6.3. Policy Recommendations Are Generic******

 The conclusion recommends "integrated environmental sustainability frameworks" and "clear monitoring and evaluation systems" (Page 13).

 These recommendations could apply to any country or region and are not tailored to the specific null finding or SSA context.

 No specific policy instrument (carbon tax, renewable energy subsidy, forest conservation payment) is named or justified.

**7. Data and Replication Issues**

******7.1. Insufficient Information for Replication******

 The paper provides source links (Page 13) but does not:

 Provide a data appendix or replication code

 Specify variable transformations (e.g., normalization min-max or z-score)

 Describe how the balanced panel was maintained over 24 years despite missing data for some countries in some years

******7.2. Time Period Justification Is Weak******

 The study covers 2000–2023, stating that 2000 is a "milestone" for global sustainability attention (Page 7).

 No evidence is provided that 2000 is a meaningful breakpoint for SSA specifically.

 The SDGs were adopted in 2015, but no analysis checks whether results differ for 2000–2014 vs. 2015–2023.

******7.3. Potential Overlap Between Predictors and Outcome******

 The sustainable development outcome likely includes environmental dimensions (e.g., SDG 13 Climate Action, SDG 15 Life on Land).

 If the EPI components (GHG emissions, renewable energy, forest cover) are also part of the SD index, this creates conceptual overlap and risks tautology.

 The paper does not address this.

**8. Presentation, Writing, and Structural Problems**

******8.1. Typographical and Factual Errors******

 Page 10: "he exctionally large" → should be "The exceptionally large"

 Page 8: "14-year period" → earlier said 24-year period (2000–2023 = 24 years)

 Page 7: "Stata Version 15" vs. Page 13: "STATA Version 16" — inconsistent

 Reference: "Bowmans" (Page 5) should be "Bouwmans" (correctly cited on Page 14)

******8.2. Redundant Text******

 Pages 3–4 contain duplicate paragraphs about global sustainability concerns and the SDGs (e.g., "The global community is currently deeply concerned..." appears twice with minor wording changes).

******8.3. Incomplete Abstract******

 The abstract (Page 1) reports results and conclusions but does not include:

 Sample size explicitly (45 countries, 1,080 observations)

 Magnitude of the insignificant effect (β = -0.0329)

 Any control variables used

**Note:** The above points must be addressed before indexing; this is a revision report on the manuscript.

Is the work clearly and accurately presented and does it cite the current literature?

Partly

If applicable, is the statistical analysis and its interpretation appropriate?

Partly

Are all the source data underlying the results available to ensure full reproducibility?

Yes

Is the study design appropriate and is the work technically sound?

Partly

Are the conclusions drawn adequately supported by the results?

Yes

Are sufficient details of methods and analysis provided to allow replication by others?

Yes

Reviewer Expertise:

Supply chain sustainability technology innovation Economics and project Management CSR

I confirm that I have read this submission and believe that I have an appropriate level of expertise to confirm that it is of an acceptable scientific standard, however I have significant reservations, as outlined above.

# Reviewer report: Reviewer response for version 1

**DOI:** 10.21956/openresafrica.17630.r34567

**Published:** 2026-05-26

**Authors:** John Kagochi

**Affiliations:** 1 Economics and Finance, Texas A&M University, Victoria, Texas, USA

The authors address an important aspect of sustainability that is critical to the Sub-Saharan African region. They use a longitudinal dataset to examine their topic by means of System GMM estimation. The authors' work demonstrates that they have produced relevant academic research and have referenced other recent studies in the field. However, there exist numerous ways for the authors to improve the paper so that it has enhanced conceptual and methodological clarity, including better theory alignment.

 One area where the authors can improve is with regard to proving clear detail on how the Environmental Practices Index (EPI) was created.  In addition to having access to publicly available data sources, the authors will also need to offer additional detail with respect to how variables were created including any normalizing, weighting, and aggregating procedures used. This will make it easier for readers to  replicate what the authors have completed. The authors should also reformulate system GMM equation and correct variable notation and format consistency. Also, although the statistical methods applied by the authors are largely accurate, they should enhance interpretation of insignificant environmental practices findings using stronger contextual explanations and theoretical interpretations.

 All in all, the authors reasonably support their conclusions based on the data which they examined. However, the paper would benefit from a more extensive analytical evaluation and more explicit policy recommendations. The revised paper should, thereafter, be should be ready for consideration for indexing in Open Research Africa.

Is the work clearly and accurately presented and does it cite the current literature?

Yes

If applicable, is the statistical analysis and its interpretation appropriate?

Yes

Are all the source data underlying the results available to ensure full reproducibility?

Partly

Is the study design appropriate and is the work technically sound?

Yes

Are the conclusions drawn adequately supported by the results?

Yes

Are sufficient details of methods and analysis provided to allow replication by others?

Partly

Reviewer Expertise:

Focus on how trade, financial development, institutions, energy infrastructure, and economic policy influence sustainable economic development and growth in Sub-Saharan Africa.

I confirm that I have read this submission and believe that I have an appropriate level of expertise to confirm that it is of an acceptable scientific standard, however I have significant reservations, as outlined above.

# Reviewer report: Reviewer response for version 1

**DOI:** 10.21956/openresafrica.17630.r34568

**Published:** 2026-05-18

**Authors:** Agnes Mutiso

**Affiliations:** 1 Kirinyaga University, Kirinyaga County, Kenya

- Generally  the article demonstrates a strong potential for indexing, It addresses a current and relevant sustainability challenges and utilizes advanced econometric methods. however a few minor issues were observed;

 -The specific environmental indicators used in constructing the environmental practices index are missing in abstract

 -policy implications and practical relevance of the study need to be included in the abstract 

 -Justify the exclusion of some countries in the methodology

 - Discussion of study limitations  is missing

Is the work clearly and accurately presented and does it cite the current literature?

Yes

If applicable, is the statistical analysis and its interpretation appropriate?

Yes

Are all the source data underlying the results available to ensure full reproducibility?

Yes

Is the study design appropriate and is the work technically sound?

Yes

Are the conclusions drawn adequately supported by the results?

Partly

Are sufficient details of methods and analysis provided to allow replication by others?

Yes

Reviewer Expertise:

I am a senior Researcher in Corporate governance and development

I confirm that I have read this submission and believe that I have an appropriate level of expertise to confirm that it is of an acceptable scientific standard, however I have significant reservations, as outlined above.

# Response

**Published:** 2026-05-24

**Authors:** JAMES C.N MBUGUA

**Affiliations:** ACCOUNTING AND FINANCE, KCA University, Nairobi, Nairobi County, Kenya

**Comment:** -The specific environmental indicators used in constructing the environmental practices index are missing in abstract

**Response:** The specific environmental indicators used in constructing the environmental practices index have been added to the abstract.

**Comment: -**Policy implications and practical relevance of the study need to be included in the abstract

**Response:** The policy implications and practical relevance of the study have been included in the abstract.

**Comment:** -Justify the exclusion of some countries in the methodology

**Response:** Thank you for your comment. We have clarified that countries with substantial missing data for key variables over the study period were excluded (Eritrea, Eswatini (formerly Swaziland), Somalia and South Sudan) to ensure a balanced and reliable panel dataset for robust System GMM estimation and valid cross-country comparability.

 - Discussion of study limitations is missing

**Response:** Study limitations have discussed as a section after conclusions

# Reviewer report: Reviewer response for version 1

**DOI:** 10.21956/openresafrica.17630.r34465

**Published:** 2026-05-13

**Authors:** Mónica Brito

**Affiliations:** 1 University of Évora, Évora, Portugal

The article addresses a topic of great relevance to Sub-Saharan Africa and to the Sustainable Development Goals (SDGs) agenda. The use of longitudinal data from 49 countries over 24 years represents a significant and potentially valuable empirical contribution to the literature on sustainability and environmental governance in emerging economies. However, the article still exhibits conceptual, methodological and structural weaknesses that limit its scientific robustness and its suitability for the standards expected by an indexed journal such as Open Research Africa.

 The article has potential merit but requires substantial revisions before it can be considered for indexing.

- **Originality and scientific relevance**

The topic is pertinent and relevant to contemporary debates on sustainability, environmental governance and African development. The regional focus on Sub-Saharan Africa is a strength, particularly as most ESG and sustainability literature remains centred on developed countries.

 However, the article does not yet clearly demonstrate what the actual research gap is; what methodological contribution it makes; how it differs from previous studies; or why the divergent results are theoretically important.

 Currently, the text reads more like a compilation of ESG literature than as innovative research with a strong academic stance.

 It is recommended that an explicit section entitled “Research Gap and Contribution” be inserted at the end of the introduction, in which the authors should explain: why previous studies fail in the African context; why the use of a dynamic panel is relevant; what this study adds theoretically to the EMT/ESG debate; and why a non-significant result is important.

- **Conceptual and theoretical issues**

The main conceptual problem with the article lies in the construction of the Environmental Practices Index (EPI). The authors do not adequately explain how the index was constructed; what weights were used; whether PCA, simple averaging or normalisation was employed; and why these three variables represent “environmental practices”.  Currently, the EPI appears more like a set of macroeconomic environmental indicators than “environmental practices”.

 The authors should explain the aggregation methodology in detail; theoretically justify why these indicators represent environmental practices, and why they are appropriate for the African context.

 EMT is presented descriptively, but is not truly operationalised in the empirical model. The authors state that technology, green innovation, and environmental regulation are central to EMT. However, none of these dimensions appear directly in the econometric model.

 There is a misalignment between the theory, the hypotheses and the variables; it is therefore recommended that the authors better align EMT with the variables used; and explain why GHG, forest cover and renewables capture EMT;

  

- **Structure and quality of writing**

Appropriate academic language is used, the formal structure is acceptable, and there is consistent use of recent literature.

 However, the introduction repeats ideas regarding the SDGs; sustainability; and economic-social-environmental balance.

 The literature review is overly descriptive, resembling a sequence of mini-summaries of articles. There is a lack of critical synthesis, conceptual comparison and analytical argumentation.

 There are some linguistic issues, such as: excessively long sentences; redundancies; terminological inconsistencies and minor grammatical errors, so a professional proofread in academic English is recommended.

  

- **Absent limitations**

The article hardly discusses any significant methodological limitations.

**Final Recommendation to the Editor**

 The manuscript addresses an important topic and has the potential to contribute to studies on sustainability in Sub-Saharan Africa. However, there are significant conceptual and methodological weaknesses, particularly regarding the construction of the Environmental Practices Index, the validity of the System GMM model, and the superficiality of the discussion of the results. A major revision is recommended before any decision on indexing.

Is the work clearly and accurately presented and does it cite the current literature?

Partly

If applicable, is the statistical analysis and its interpretation appropriate?

I cannot comment. A qualified statistician is required.

Are all the source data underlying the results available to ensure full reproducibility?

Partly

Is the study design appropriate and is the work technically sound?

Partly

Are the conclusions drawn adequately supported by the results?

Partly

Are sufficient details of methods and analysis provided to allow replication by others?

No

Reviewer Expertise:

Sustainable Development; Tourism; Tourism planning and development

I confirm that I have read this submission and believe that I have an appropriate level of expertise to state that I do not consider it to be of an acceptable scientific standard, for reasons outlined above.

# Response

**Published:** 2026-05-24

**Authors:** JAMES C.N MBUGUA

**Affiliations:** ACCOUNTING AND FINANCE, KCA University, Nairobi, Nairobi County, Kenya

**Comment: Originality and scientific relevance** The topic is pertinent and relevant to contemporary debates on sustainability, environmental governance and African development. The regional focus on Sub-Saharan Africa is a strength, particularly as most ESG and sustainability literature remains centred on developed countries.

 However, the article does not yet clearly demonstrate what the actual research gap is; what methodological contribution it makes; how it differs from previous studies; or why the divergent results are theoretically important.

 Currently, the text reads more like a compilation of ESG literature than as innovative research with a strong academic stance.

 It is recommended that an explicit section entitled “Research Gap and Contribution” be inserted at the end of the introduction, in which the authors should explain: why previous studies fail in the African context; why the use of a dynamic panel is relevant; what this study adds theoretically to the EMT/ESG debate; and why a non-significant result is important. **Response:** Thank you for your comment. We have revised the manuscript to explicitly clarify the research gap and contribution by adding a dedicated section under Research Gap and Contribution. In this addition, we highlight the empirical gap arising from limited Sub-Saharan Africa-focused studies, the methodological gap addressed through the use of a dynamic System GMM approach, and the theoretical extension to the Ecological Modernization Theory (EMT) and ESG literature in a context of weak institutional capacity. In addition, we have expanded the discussion of the non-significant findings by providing a more critical interpretation grounded in Ecological Modernization Theory, emphasizing that Sub-Saharan Africa remains at an early or incomplete stage of ecological modernization. We further explain that weak institutional capacity, fragmented policy implementation, limited funding, governance challenges, and weak enforcement mechanisms constrain the effectiveness of environmental practices and limit their ability to generate measurable sustainable development outcomes.  

**Comment:** **Conceptual and theoretical issues** The main conceptual problem with the article lies in the construction of the Environmental Practices Index (EPI). The authors do not adequately explain how the index was constructed; what weights were used; whether PCA, simple averaging or normalisation was employed; and why these three variables represent “environmental practices”.  Currently, the EPI appears more like a set of macroeconomic environmental indicators than “environmental practices”.

 The authors should explain the aggregation methodology in detail; theoretically justify why these indicators represent environmental practices, and why they are appropriate for the African context.

 EMT is presented descriptively, but is not truly operationalised in the empirical model. **Response:  **Thank you for your comment. We appreciate the concern regarding the construction and conceptual grounding of the Environmental Practices Index (EPI) and its alignment with Ecological Modernization Theory (EMT). In response, we have already addressed this issue in the revised manuscript by providing a detailed explanation of the EPI construction, including its components, normalization process, and aggregation approach, as well as the justification for why these indicators appropriately capture environmental practices in the Sub-Saharan African context. In addition, we have strengthened the theoretical operationalization of EMT by explicitly linking the selected environmental indicators to key EMT dimensions such as resource efficiency, decoupling of growth from environmental degradation, and transition toward low-carbon development. These revisions ensure clearer conceptual clarity, stronger theoretical grounding, and improved alignment between the empirical model and the underlying EMT framework.     The authors state that technology, green innovation, and environmental regulation are central to EMT. However, none of these dimensions appear directly in the econometric model.

**Comment: **There is a misalignment between the theory, the hypotheses and the variables; it is therefore recommended that the authors better align EMT with the variables used; and explain why GHG, forest cover and renewables capture EMT;

**Response:** Thank you for your insightful comment. We have revised the theoretical framework to better align Ecological Modernization Theory (EMT) with the empirical model by explicitly explaining how greenhouse gas emissions, renewable energy consumption, and forest cover serve as observable proxies of key EMT dimensions, including decoupling, technological transition, and ecological conservation. We also clarify that direct measures of technological innovation and regulatory stringency are limited in cross-country Sub-Saharan African panel data, necessitating the use of validated environmental outcome indicators to operationalise EMT in the empirical analysis.

**Comment:** **Structure and quality of writing** Appropriate academic language is used, the formal structure is acceptable, and there is consistent use of recent literature.

 However, the introduction repeats ideas regarding the SDGs; sustainability; and economic-social-environmental balance.  

**Response:** Thank you for your insightful comment. We have revised the background of the study by removing repetitive statements on the SDGs and the general definition of sustainable development, particularly those related to the triple-bottom-line framework. This has improved the logical flow, reduced redundancy, and strengthened the focus of the introduction on the Sub-Saharan African context and environmental practices literature.

**Comment: **The literature review is overly descriptive, resembling a sequence of mini-summaries of articles. There is a lack of critical synthesis, conceptual comparison and analytical argumentation.

 There are some linguistic issues, such as: excessively long sentences; redundancies; terminological inconsistencies and minor grammatical errors, so a professional proofread in academic English is recommended.

**Response: **Thank you for your valuable comment. We have revised the literature review to improve analytical depth by reducing descriptive summaries, removing redundancies, and strengthening conceptual synthesis across studies. The revised section now organizes the literature thematically and provides a more critical comparison of environmental governance, firm-level environmental practices, and macroeconomic sustainability outcomes. We have also improved coherence by addressing terminological inconsistencies, reducing overly long sentences, and enhancing academic clarity.

**Comment:** **Absent limitations** The article hardly discusses any significant methodological limitations.  

**Response: **Methodological limitations have discussed as a section after conclusions.

# Reviewer report: Reviewer response for version 1

**DOI:** 10.21956/openresafrica.17630.r34128

**Published:** 2026-04-15

**Authors:** Ivon Arisanti

**Affiliations:** 1 Universitas Teknologi Sumbawa, Sumbawa Besar, Indonesia

Overall, this manuscript addresses a topic of considerable importance and has the potential to contribute meaningfully to the literature, particularly given its empirical focus on Sub-Saharan Africa. The findings are interesting and may offer a valuable perspective, especially where they diverge from commonly expected results. At the same time, several areas require further clarification and strengthening before the manuscript can meet the standards expected for indexing. I would encourage the authors to carefully consider the points raised. With substantial revision and further development, the manuscript has the potential to become a valuable contribution to the field and may be suitable for indexing in due course.

 In particular, the authors are encouraged to consider the following points:

 1. Model validity (System GMM): The current results suggest a possible issue related to instrument proliferation (e.g., the Hansen test p-value). It would be advantageous to revisit the instrument strategy, potentially by reducing the number of instruments and providing additional justification for their selection.           

 2. Clarification of the main variable (EPI):  The construction of the Environmental Practices Index would benefit from a more detailed explanation, including its components, data sources, and aggregation approach, to enhance clarity and transparency

 3. Articulation of research gaps and contributions:

 The manuscript would be improved by more clearly highlighting its specific contribution to the literature, particularly in relation to existing empirical and methodological gaps in the Sub-Saharan African context.

 4. Depth of discussion:

 The discussion of the non-significant findings could be further developed, especially by engaging more critically with possible contextual and theoretical explanations.

Is the work clearly and accurately presented and does it cite the current literature?

Partly

If applicable, is the statistical analysis and its interpretation appropriate?

Partly

Are all the source data underlying the results available to ensure full reproducibility?

Partly

Is the study design appropriate and is the work technically sound?

Partly

Are the conclusions drawn adequately supported by the results?

Partly

Are sufficient details of methods and analysis provided to allow replication by others?

Partly

Reviewer Expertise:

Focus on Human Resource Management and Gender, which is relevant to social/organisational justice and impacts organizational performance

I confirm that I have read this submission and believe that I have an appropriate level of expertise to state that I do not consider it to be of an acceptable scientific standard, for reasons outlined above.

# Response

**Published:** 2026-05-24

**Authors:** JAMES C.N MBUGUA

**Affiliations:** ACCOUNTING AND FINANCE, KCA University, Nairobi, Nairobi County, Kenya

**Comment: **1. *Model validity (System GMM): The current results suggest a possible issue related to instrument proliferation (e.g., the Hansen test p-value). It would be advantageous to revisit the instrument strategy, potentially by reducing the number of instruments and providing additional justification for their selection.  *         

**Response: **We agree with this comment. We have revisited the instrument strategy by limiting the instrument count to 38 (below the number of cross-sectional units, N=45) and refining the lag structure to a maximum of two lags, thereby minimizing instrument proliferation while maintaining model efficiency. The revised Hansen J-test yields χ² = 28.54 (df = 22, p = 0.156) and the Difference-in-Hansen test yields χ² = 8.67 (df = 10, p = 0.564), confirming instrument validity without evidence of overfitting. Additional justification for instrument selection has been incorporated into the methodology and results discussion sections.

**Comment: **2. Clarification of the main variable (EPI):  The construction of the Environmental Practices Index would benefit from a more detailed explanation, including its components, data sources, and aggregation approach, to enhance clarity and transparency.

**Response: **Thank you for your comment. We have added a detailed paragraph clarifying the construction of the Environmental Practices Index, including its components, data sources, normalization process, and aggregation method using equal weighting to enhance transparency and replicability of the study.

**Comment: **3. Articulation of research gaps and contributions:

 The manuscript would be improved by more clearly highlighting its specific contribution to the literature, particularly in relation to existing empirical and methodological gaps in the Sub-Saharan African context.

**Response:** Thank you for your comment. We have revised the manuscript and added a section on Research Gap and Contribution to clearly articulate the study’s specific contributions by explicitly highlighting the empirical, methodological, and contextual gaps addressed. In particular, we have emphasized the use of a large panel dataset for Sub-Saharan Africa, the application of System GMM for robust estimation, and the integration of multiple sustainability dimensions within a unified framework.

**Comment: **4. Depth of discussion:

 The discussion of the non-significant findings could be further developed, especially by engaging more critically with possible contextual and theoretical explanations.

**Response:** Thank you for your comment. We have expanded the discussion of the non-significant findings by providing a more critical interpretation grounded in Ecological Modernization Theory, highlighting that Sub-Saharan Africa is still at an early or incomplete stage of ecological modernization. We further explain that weak institutional capacity, fragmented policy implementation, limited funding, governance challenges, and weak enforcement mechanisms constrain the effectiveness of environmental practices and limit their ability to generate measurable sustainable development outcomes.
